# Modeling Hepatitis C Elimination Among People Who Inject Drugs in New Hampshire

**DOI:** 10.1001/jamanetworkopen.2021.19092

**Published:** 2021-08-03

**Authors:** Andrew Blake, James E. Smith

**Affiliations:** 1 Brigham and Women's Hospital, Boston, Massachusetts; 2 Tuck School of Business at Dartmouth, Hanover, New Hampshire

## Abstract

**Importance:**

The success of direct-acting antiviral therapies for chronic hepatitis C virus (HCV) infection led the World Health Organization to set elimination targets by 2030. For the United States to achieve these benchmarks, public health responses must target high-risk populations, such as people who inject drugs (PWID), a group with high rates of HCV incidence and low rates of treatment uptake.

**Objective:**

To evaluate potential improvements in the HCV care cascade among PWID, focusing on improved testing, treatment uptake, and access to harm reduction.

**Design, Setting, and Participants:**

This decision analytic model used a differential equation–based dynamic transmission model based on data from New Hampshire, an illustrative state with a large number of PWID and limited HCV treatment infrastructure. Surveillance data through 2020 was used for model parameterization, and the final analysis was conducted in May 2021.

**Main Outcomes and Measures:**

Model forecasts of chronic HCV cases and advanced-stage HCV outcomes from 2022 to 2045.

**Results:**

A total of 6 scenarios were tested: (1) the base case, (2) improved harm reduction, (3) improved testing, (4) improved treatment, (5) improved testing and treatment, and (6) improved testing, treatment, and harm reduction. All scenarios with improved testing, treatment uptake, and/or access to harm reduction were associated with decreases in forecasted HCV prevalence and HCV-associated mortality compared with the base case. Improving harm reduction, testing, and treatment individually were forecast to reduce prevalence of HCV in 2045 from 69.7% in the base case to 62.8%, 45.7%, and 35.5%, respectively. Combining treatment and testing improvements was associated with a 2045 prevalence of 0.3%; adding harm reduction improvements was associated with further reductions in prevalence forecasts (to 0.2%), with fewer total treatments (10 960 vs 13 219 from 2022-2045).

**Conclusions and Relevance:**

In this modeling study, no single intervention was projected to achieve World Health Organization HCV elimination targets. Scenarios with improvements in both testing and treatment were associated with a prevalence of less than 3% by 2030 and achieved elimination targets. Adding improvements in harm reduction was associated with faster reductions in prevalence and fewer treatments.

## Key Points

**Question:**

What improvements in the hepatitis C (HCV) care cascade are required to eliminate HCV among people who inject drugs (PWID)?

**Findings:**

This decision analytic model of HCV transmission found that improved testing, treatment, and access to harm reduction were all associated with reductions in HCV prevalence and mortality among PWID. Improvements in both testing and treatment were associated with HCV prevalence of less than 2% by 2030.

**Meaning:**

These findings suggest that HCV elimination may be possible among PWID by 2030 with improved testing and treatment; improved harm reduction may reduce the time and number of treatments required to achieve similar outcomes.

## Teaser

This decision analytical model evaluates potential improvements in the hepatitis C virus (HCV) care cascade among people who inject drugs (PWID), focusing on improved testing, treatment uptake, and access to harm reduction.

## Introduction

An estimated 2.4 million people in the United States live with chronic hepatitis C virus (HCV), and HCV-associated deaths are higher than the next 60 reportable infectious diseases combined.^1,2^ The development of direct-acting antivirals has dramatically improved our ability to treat HCV, and the World Health Organization (WHO) proposed 2030 HCV elimination targets, including an 80% reduction in new chronic infections and a 65% reduction in mortality from 2015 levels.^3^

Despite this progress, incident HCV cases continue to increase in the United States.^4^ The continued spread of HCV is primarily a consequence of injection drug use; injection-related transmission is now responsible for most new HCV cases.^5^ Thus, to reach WHO goals, treating people who inject drugs (PWID) is a priority.

Previous studies have identified barriers that PWID face in the HCV care cascade, including low screening rates, low treatment uptake, and ongoing risk of reinfection.^6,7,8,9,10^ Interventions aimed at addressing these issues include improved HCV screening, improvements in keeping patients linked to care, and improved access to harm reduction services, such as syringe service programs (SSPs) and medication-assisted treatment (MAT).^11,12^ These interventions are typically implemented at the state level, and state policies on HCV surveillance, Medicaid reimbursement, and access to harm reduction may affect the success of these efforts.^13,14,15^

We developed a mathematical model to evaluate improvements to the PWID HCV care cascade (ie, improved testing, improved treatment uptake, and improved access to harm reduction services) and forecast HCV outcomes associated with these interventions. We used New Hampshire as an illustrative setting because it has high rates of injection drug use and, like many states, has underdeveloped infrastructure to track and treat HCV among PWID (eAppendix and eFigure 1 in the Supplement).^16,17^ To our knowledge, previous studies^12,18,19^ of HCV elimination among PWID have not modeled the full range of interventions considered here.

## Methods

### Model Overview

We developed a dynamic, compartmental, differential equation model to simulate the spread of HCV among PWID. Differential equation models have been widely applied to the study of HCV, and our analysis follows previously established guidelines from the Good Research Practices in Modeling Task Force.^20^ The model has a total of 3456 compartments representing all possible combinations of the PWID classifications described in the Population Submodel section. The number of PWID in each compartment is tracked over time, and the transitions among these compartments are governed by a differential equation.

In the model, the PWID population is assumed to be in a steady state before 2013, with little testing for or treatment of HCV. The period from 2013 to 2022 includes the opioid crisis and the arrival of directing-acting antivirals to treat HCV. Forecasts are generated from 2022 through 2045 in scenarios described on the Statistical Analysis section. This forecast horizon extends 15 years longer than the 2030 WHO goals, and the long-term behavior of the model is clear by 2045.

In this section, we provide a high-level overview of the model and key parameters; additional details on the model appear in the eAppendix, eFigure 2, and eFigure 3 in the Supplement; the data sources and model validation appear in the eAppendix in the Supplement. Parameters were selected to represent the situation in New Hampshire and are based on data from the National Surveys on Drug Use and Health, state reporting, and available literature.^21,22,23,24,25,26,27^ We used data through 2020 when available; final analysis was conducted in May 2021. The Common Rule exempts this study from institutional board review because no human participants were involved.

### Population Submodels

PWID enter the model at the initiation of their use of injection drugs and are categorized along dimensions that predict risky or protective behaviors. These dimensions are as follows: (1) injecting status, ie, recent (those who been injecting <5 years), nonrecent (5-8 years), long-term (>8 years), and inactive,^28,29^; (2) comorbid use of stimulants (eg, cocaine or amphetamines) and opioids (eg, heroin or fentanyl), yes or no; and (3) participation in MAT and/or SSPs, yes or no for each.^28,30^

In the model, people transition among these categories. For example, active PWID age and move from recent to nonrecent and long-term categories. Active PWID may cease injecting and become inactive, and inactive PWID may relapse and resume injecting drugs; we assumed cessation and relapse rates based on published data.^31^ Similarly, people may start or leave MAT or SSPs. In the base-case scenario, we assumed 15% of active PWID are currently participating in SSP and 15% are receiving MAT; 17% of the population has comorbid stimulant and opioid use.^24,32,33^ People exit the model through background mortality, with higher mortality rates for active PWID.

Based on the National Surveys on Drug Use and Health and state overdose data, we assumed that there were 8000 active PWID in New Hampshire in 2018, representing a 2-fold increase in the population of active PWID from 2013.^12,26,34^ After 2018, we assumed a dampening of the opioid epidemic with the population of active PWID declining to 90% of the 2019 peak by 2022.^22,26,27^ The new PWID inflow rates in the model were different in the pre-2013, 2013 to 2018, and 2018 to 2045 periods and are calibrated to match these population estimates.

### Infection Submodel

Entering PWID are assumed to be susceptible to HCV, and active PWID are infected at a rate proportional to the current HCV prevalence and depending on the risk factors described previously. For example, PWID with comorbid opioid and stimulant use are 2.13 times more likely (all else equal) to acquire HCV than others.^28^ Recent PWID are 2.60 times more likely to become infected than long-term PWID.^29^ PWID enrolled in MAT and SSP are 50% and 56% less likely, respectively, to acquire an infection than those not enrolled in these harm-reduction programs.^12^

The overall force of infection was calibrated to match HCV prevalence based on New Hampshire HCV surveillance data.^21,25^ Specifically, we assume the prevalence was 40% in 2013 and 45% in 2019.^21,35^ The 2013 to 2022 infection rate is assumed to continue into the 2022 to 2045 forecast period.^12^

### Liver Disease Submodel

**Figure 1.**
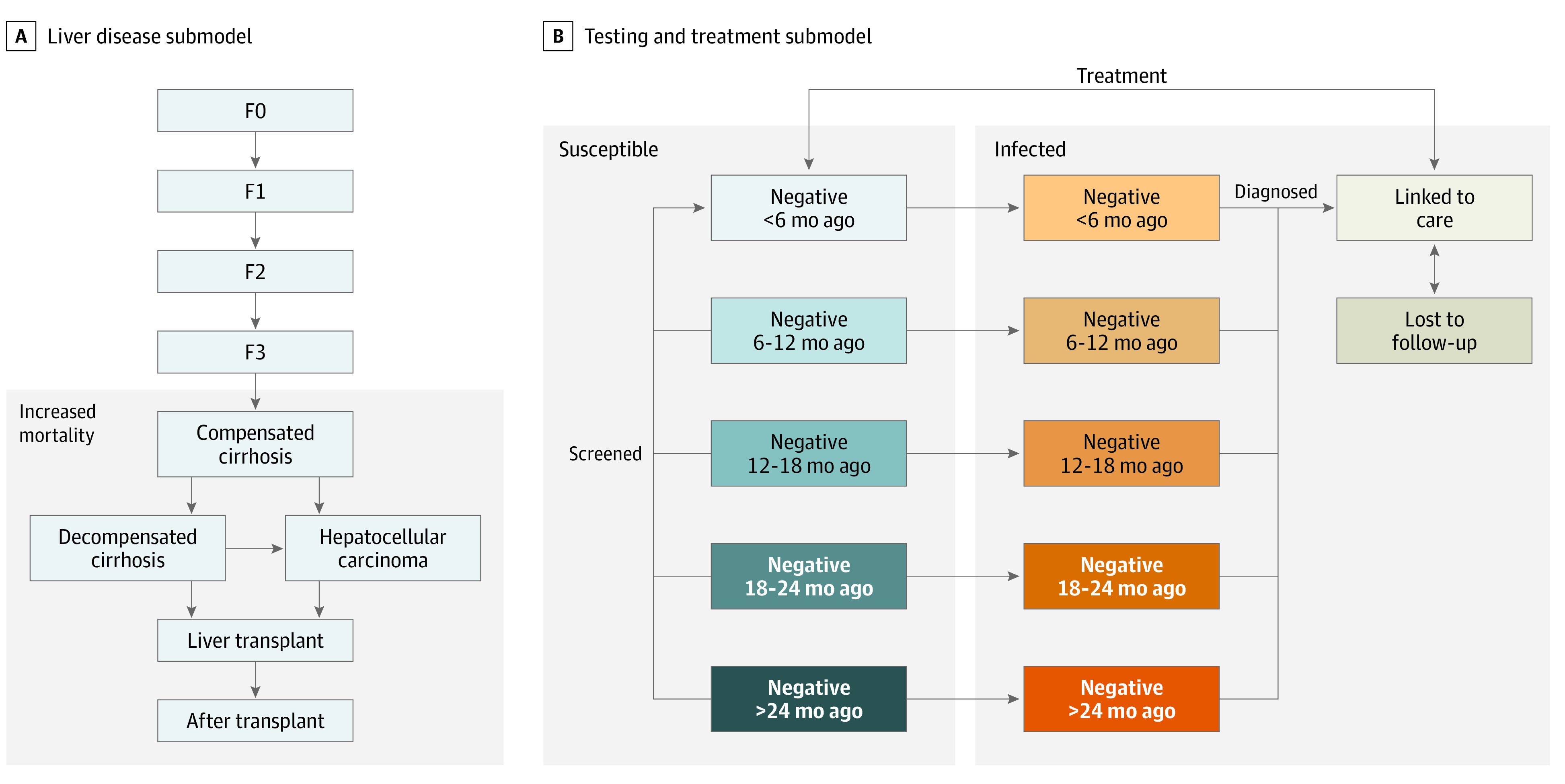
Selected Submodels

PWID who have chronic infection progress through stages of liver disease, which are categorized using the METAVIR scoring system (Figure 1A). Individuals with infection and no prior history of liver disease begin with no fibrosis (F0) and advance through subsequent METAVIR stages. PWID with cirrhosis (F4) may progress to decompensated cirrhosis and/or hepatocellular carcinoma and may receive a liver transplant. People with advanced liver disease have increased mortality risks. Our parameters are based on similar models of liver disease and assume that treating HCV stops progression through the F0 to F4 stages and slows progression at later stages but does not reverse liver damage.^36^

### Testing and Treatment Submodel

Patients are diagnosed at rates that depend on their liver disease state, their participation in MAT or SSPs, and the time since the patient was last known to test negative for HCV (Figure 1B).^37^ Tracking the time since a patient was last known to test negative allows us to distinguish between policies with testing at scheduled intervals (eg, patients receiving regular care and screening at SSPs and MATs) and those with irregular testing. For example, a patient receiving annual testing as part of an MAT program has a high chance of being tested if the last negative test result was more than 12 months ago.

The model assumes that entering PWID are known to not have HCV. As shown in Figure 1B, a negative test result resets the time since the last negative result. A positive test result leads to a patient being linked to care. Patients who are linked to care are lost to follow-up or treated. Individuals receiving treatment achieve sustained viral response with 95% probability and reenter the susceptible population with no immunologic protection against reinfection. Those who are lost to follow-up are reengaged at rates that depend on their testing rate.

The testing and treatment rates vary across scenarios. In the base case, we assume PWID who are asymptomatic (either uninfected or having METAVIR stages F0-F3) are diagnosed at a rate of once per 20 years.^18^ Patients who are symptomatic for liver disease (METAVIR stage ≥F4) are diagnosed at higher rates. Treatment rates were calibrated to New Hampshire surveillance data for PWID and assume that 20% of those diagnosed complete treatment.^21^

### Statistical Analysis

We considered 6 model scenarios and performed 3 sensitivity analyses. Calculations were performed in MATLAB R2019b (MathWorks).

#### Model Scenarios

We consider a variety of potential policy scenarios related to improving testing, treatment, and harm reduction. Specifically, we focus on forecasts of HCV prevalence, incidence, treatments, and liver deaths through 2045 in the following 6 scenarios:

1. Base case: we assume 15% of active PWID are in an SSP and 15% of active PWID are receiving MAT. Asymptomatic people (METAVIR scores F0-F3) are diagnosed with chronic HCV at a rate of once per 20 years^18,25^; 20% of those diagnosed with chronic HCV are treated.^21^
2. Improved harm reduction: this scenario takes the base case but increases enrollment in SSP and MAT. In this scenario, 50% of active PWID are in an SSP, and 35% of active PWID are receiving MAT.
3. Improved testing: this scenario takes the base case but increases testing. Testing in primary care and emergency departments increases to once per 2 years (on average), and testing for those receiving MAT and SSP increased to once per year.
4. Improved treatment: this scenario takes the base case but removes attrition in the HCV treatment process. For example, treatment takes place at diagnosis.
5. Improved testing and treatment: this scenario combines the testing and treatment interventions from scenarios 3 and 4.
6. Improved testing, treatment, and harm reduction: this scenario combines all 3 interventions from scenarios 2, 3, and 4.

#### Sensitivity Analysis

In addition, we consider 3 additional sensitivity analyses. First, the 6 scenarios described previously consider certain combinations of 4 dimensions of possible interventions (ie, increased testing in the primary care and emergency departments, annual testing in MAT/SSP, increased treatment uptake, and increased harm reduction enrollment). To better understand the interactions among these dimensions, we evaluated all possible combinations of these interventions, considering aggressive improvements (as in the scenarios previously described) as well as intermediate cases. Second, given the lack of precise data on PWID, we conducted a Monte Carlo simulation in which the parameters were considered uncertain to better understand the robustness of the results. The full details of the Monte Carlo simulation are described in the eAppendix in the Supplement. Third, because the cost of direct-acting antivirals is a possible barrier to widespread treatment, we consider limits on the treatment rate in scenario 6. In these cases, treatments were prorated among those eligible for treatment to meet a given limit.

## Results

**Figure 2.**
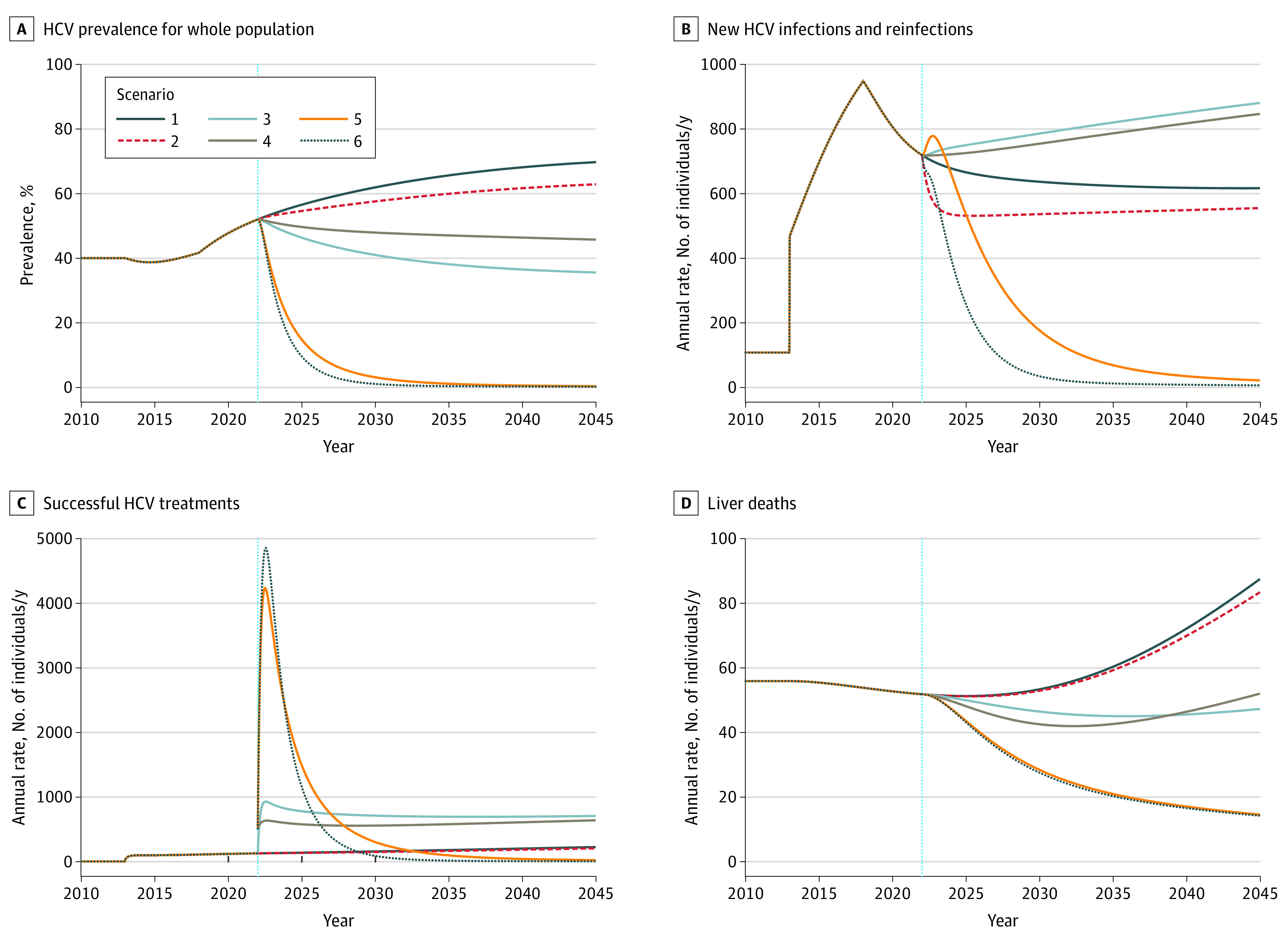
Scenario Results HCV indicates hepatitis C virus. Scenario 1 is the base case; scenario 2, improved harm reduction; scenario 3, improved testing; scenario 4, improved treatment; scenario 5, improved testing and treatment; scenario 6, improved testing, treatment, and harm reduction. The vertical blue line indicates when the model was run.

Figure 2 shows model forecasts of HCV prevalence, incidence, treatments, and HCV-related liver deaths through 2045 for the 6 scenarios described above; key numerical results are summarized in the Table. Detailed results for each scenario are provided in eFigure 4 to eFigure 9 in the Supplement.

**Table.** Summary of Model Forecasts

Scenario	HCV prevalence in 2045, %	Total No., 2022-2045			
		HCV infections	Cirrhosis cases	HCV-related deaths	HCV treatments
1. Base case	69.7	14 638	3674	1424	4021
2. Improved harm reduction	62.8	12 540	3506	1395	3763
3. Improved testing	35.5	18 570	1887	1079	16 650
4. Improved treatment	45.7	17 879	2601	1053	13 564
5. Improved testing and treatment	0.3	4619	203	616	13 219
6. Improved testing, treatment, and harm reduction	0.2	2173	165	603	10 960

Abbreviation: HCV, hepatitis C virus.

The forecasts for all interventions show decreases in HCV prevalence and liver deaths compared with the base case (scenario 1). Improving harm reduction, testing, and treatment individually was associated with 2045 prevalences of 62.8%, 45.7%, and 35.5% respectively, compared with 69.7% in the base case. The 2 scenarios that include both testing and treatment improvements (scenarios 5 and 6) were forecast to have near-zero HCV prevalence by 2045 (0.3% and 0.2%) (Figure 2A). These 2 scenarios had many HCV treatments early in the forecast period but required fewer total treatments than improving testing or treatment alone (Figure 2C). In scenarios 5 and 6, the rapid reduction in prevalence associated with the high initial treatment rates led to fewer infections (Figure 2B) and treatments later.

Scenarios 5 and 6 were also forecast to have fewer new cases of compensated cirrhosis (203 and 165 cases respectively) and liver deaths (616 and 603 cases respectively) (Table) compared with all other scenarios. In contrast, the other scenarios show annual deaths rising (scenarios 1 and 2) or changing little (scenarios 3 and 4) over the forecast period. The forecast reductions in deaths in scenarios 5 and 6 were less pronounced than the reductions in incidence, prevalence, and new cirrhosis cases because most of the forecast deaths in these scenarios occur among those who have advanced liver disease in 2022, which is assumed to be irreversible in the model.

**Figure 3.**
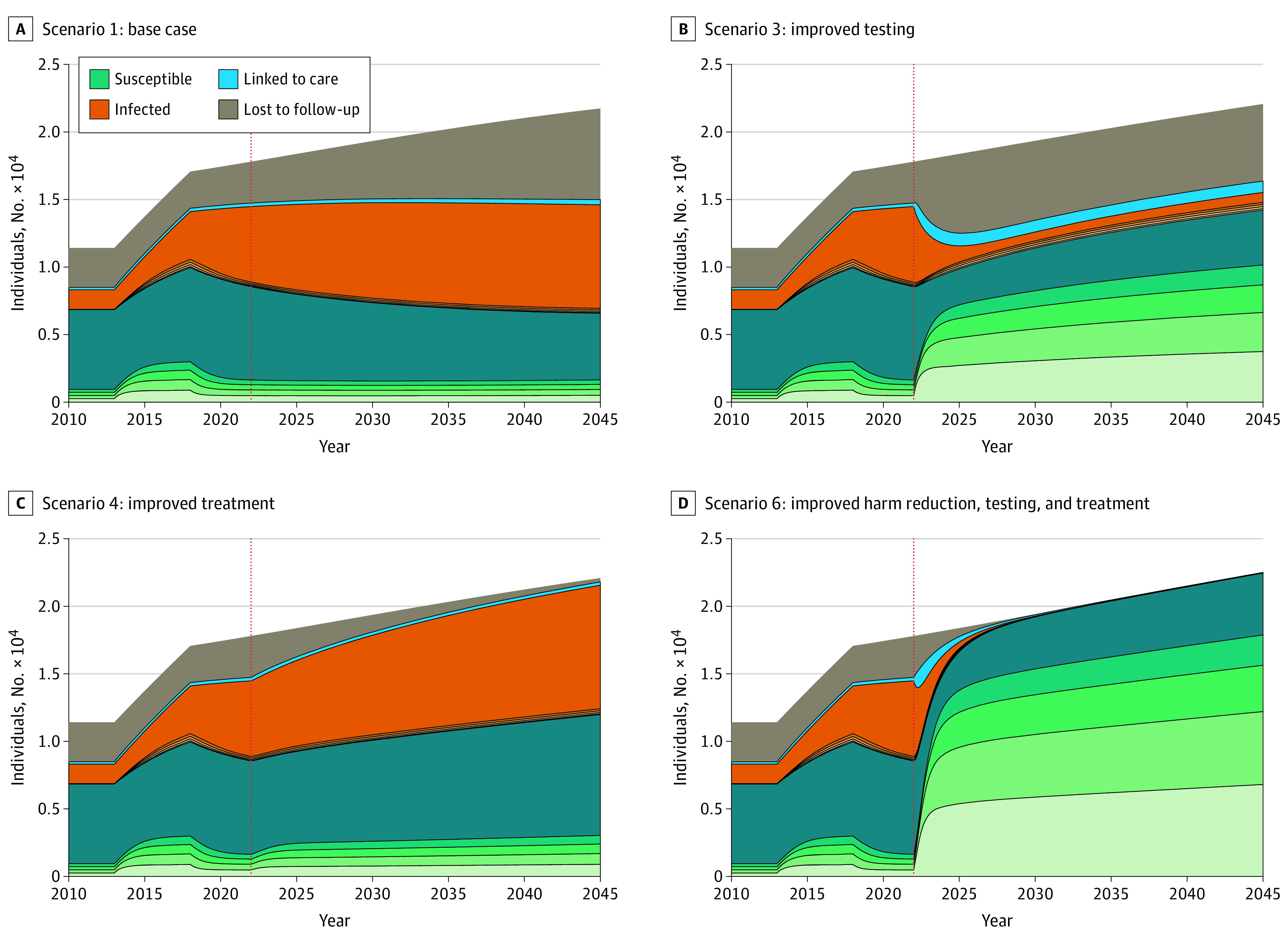
Testing and Treatment Populations Across Select Scenarios

Figure 3 shows where PWID are in the testing and treatment process over time. In the base case, most people, those with infection and those without, have not received testing within the last 24 months; thus, testing is a bottleneck in the care cascade. In scenario 3 (Figure 3B), there are fewer individuals with undiagnosed infections, but more patients were lost in the treatment process. In scenario 4 (Figure 3C), PWID with HCV were successfully treated, but the bottleneck in testing remained, as in the base case. In scenario 6 with improvements in testing, treatment, and harm reduction (Figure 3D), most of the population had been tested in the last year, and those who tested positive were promptly treated.

In the model, harm-reduction programs reduced infection among active PWID in 2 ways. First, harm-reduction programs reduce transmission by reducing injection and/or sharing of needles; among single interventions, improving harm-reduction was forecast to have the largest reduction in the number of infections (Figure 2B). Second, harm-reduction provides annual testing for those enrolled in the programs in scenarios 3, 5, and 6. Consequently, scenario 6 (with all improvements) was forecast to have a more rapid decrease in prevalence than scenario 5 (without harm prevention), with fewer new infections and treatments (2173 vs 4619 new infections and 10 960 vs 13 219 treatments).

### Sensitivity Analyses

In the Supplement, we present results for 3 additional sensitivity analyses described previously. When considering the results of all possible combinations of interventions (eFigure 10 in the Supplement), we observed that the interventions were synergistic. For example, the benefits associated with annual testing in MAT and SSP are greater when pursued with other interventions. Of the 72 scenarios considered, the lowest prevalence in 2045 (0.2%) was associated with pursuing all of the interventions aggressively, as in scenario 6; this scenario also had fewer treatments required than any other scenario that has a 2045 prevalence of less than 40%.

In the Monte Carlo simulation, in which model parameters were considered uncertain, we found that the qualitative conclusions were quite robust (eFigure 11 to eFigure 16 in the Supplement). Although the specific results vary across simulation trials, scenario 6 (which includes improvements in testing, treatment, and harm reduction) was consistently associated with near-zero HCV prevalence by 2045 (10th and 90th percentiles of 0.0% and 0.3%) and fewer treatments than scenario 5; scenarios 1 to 4 did not achieve near-zero prevalence for any combination of parameter values. However, there is considerable uncertainty in the number of treatments required (10th and 90th percentiles of 8179 and 13 950, respectively, in the forecast period for scenario 6), reflecting the uncertainty regarding the number of PWID with HCV in the current (ie, 2022) population.

If we limit the treatment rate (reflecting possible resource or budget constraints in scenario 6), the forecasted decrease in prevalence was slower, and the total number of treatments increased. However, if the limit on the annual treatment rate is 1000 or greater, the forecast prevalence was near-zero by the early 2030s (eFigure 17 in the Supplement).

## Discussion

We developed a dynamic model to evaluate interventions aimed at specific failures in the HCV care cascade for PWID, ie, insufficient testing, low treatment uptake, and inadequate access to harm reduction, and forecasted HCV outcomes associated with these interventions in New Hampshire. Our results suggest that improvements in both testing and treatment are required to achieve the WHO goal of reducing HCV incidence by 80% among PWID in New Hampshire by 2030. Monte Carlo simulation suggests this conclusion is robust despite parameter uncertainty. However, achieving similar reductions in HCV-related mortality by 2030 is unlikely because of the many PWID who currently have advanced liver disease. If we were to assume that HCV treatments can reverse liver damage in patients with advanced fibrosis, these mortality forecasts might be improved.^38^

Although other states may experience different bottlenecks than New Hampshire, studies have consistently found insufficient testing and poor rates of treatment after diagnosis among PWID.^7,8,9,21^ Thus, similar improvements in testing and treatment may be required to reach elimination targets in other states. Bottlenecks in the HCV care cascade often originate from state-level policies on Medicaid reimbursement and HCV surveillance efforts. Examples of interventions to increase testing and treatment uptake include conducting screening and treatment in prisons; community integration of HCV education, workup, and treatment; and removing Medicaid restrictions on sobriety, prescriber, and fibrosis criteria.^11,13,15,39^ The lack of good state-level data on PWID poses a challenge for policy makers seeking to identify and address weaknesses in their HCV care cascade; resources like the Hepatitis C Medicaid Affinity Group can help states share best practices.^40^

Our results also highlight an important role played by harm-reduction programs. In addition to reducing the risk of infection, harm-reduction programs can promote regular testing, facilitate linkage to care, and provide access to active PWID who are not often reached in traditional settings. Despite the well-documented benefits of harm-reduction programs, access to both MAT and SSPs remains limited and varies widely by state.^41,42,43,44^ Our analysis suggests that increases in the availability of harm-reduction services could enhance testing and treatment efforts and may reduce the number of treatments required for HCV elimination.

The cost associated with treatment is likely to be a key concern for state stakeholders, particularly given the high cost of direct-acting antivirals and the high prevalence of HCV among Medicaid beneficiaries.^15,45^ The scenarios associated with HCV elimination are forecast to require a substantial increase in treatments before 2030 but fewer total treatments over the forecast period. Payment models in which a state pays a subscription fee for unlimited HCV treatments over a fixed period (ie, the Netflix model) may be well suited for the early years of an elimination effort.^46^

### Limitations

There are several limitations to this study. As in other studies of HCV among PWID, the data necessary to parameterize the model are scarce, particularly at the state level. We used New Hampshire–specific data when possible and included wide ranges of uncertainty for parameters in our Monte Carlo simulation analysis. We did not model HCV among other high-risk groups besides PWID, notably baby boomers, incarcerated populations, and men who have sex with men.^47^ We did not consider the effects of HIV-HCV coinfection, which can result in a more severe disease course.^48^ In addition, we did not conduct a cost-effectiveness analysis of the interventions considered here; such a study would be useful to better understand the tradeoffs involved.

## Conclusions

In this modeling study, no single intervention was projected to achieve World Health Organization HCV elimination targets. However, the findings suggest that coordinated improvements in testing, treatment, and harm-reduction programs could greatly reduce the HCV burden among PWID in New Hampshire and other states facing similar challenges.

## Notes

Supplement. **eAppendix. **Supplementary Methods and Results eReferences. **eFigure 1.** Drug Overdose Rates **eFigure 2.** Injector Duration Submodel Structure **eFigure 3.** MAT/SSP Enrollment Submodel Structure **eFigure 4.** Model Forecasts for Scenario 1, Base Case **eFigure 5.** Model Forecasts for Scenario 2, Improved Harm Reduction **eFigure 6.** Model Forecasts for Scenario 3, Improved Testing **eFigure 7.** Model Forecasts for Scenario 4, Improved Treatment **eFigure 8.** Model Forecasts for Scenario 5, Improved Testing and Treatment **eFigure 9.** Model Forecasts for Scenario 6, Improved Testing, Treatment and Harm Reduction **eFigure 10.** Policy Intervention Sensitivity Analysis **eFigure 11.** Simulation Results for Scenario 1, Base Case **eFigure 12.** Simulation Results for Scenario 2, Increased Harm Reduction **eFigure 13.** Simulation Results for Scenario 3, Improved Testing **eFigure 14.** Simulation Results for Scenario 4, Improved Treatment **eFigure 15.** Simulation Results for Scenario 5, Improved Testing and Treatment **eFigure 16.** Simulation Results for Scenario 6, Improved Testing, Treatment, and Harm Reduction **eFigure 17.** Sensitivity Analysis to Limit on Treatment Rate in Scenario 6
