# Supplementary material for: Modeling Hepatitis C Elimination Among People Who Inject Drugs in New Hampshire
Source: JAMA Netw Open. 2021 Aug 3;4(8):e2119092. doi: 10.1001/jamanetworkopen.2021.19092 (PMC8335578; doi:10.1001/jamanetworkopen.2021.19092)
Supplement: Supplement. — eAppendix. Supplementary Methods and Results eReferences. eFigure 1. Drug Overdose Rates eFigure 2. Injector Duration Submodel Structure eFigure 3. MAT/SSP Enrollment Submodel Structure eFigure 4. Model Forecasts for Scenario 1, Base Case eFigure 5. Model Forecasts for Scenario 2, Improved Harm Reduction eFigure 6. Model Forecasts for Scenario 3, Improved Testing eFigure 7. Model Forecasts for Scenario 4, Improved Treatment eFigure 8. Model Forecasts for Scenario 5, Improved Testing and Treatment eFigure 9. Model Forecasts for Scenario 6, Improved Testing, Treatment and Harm Reduction eFigure 10. Policy Intervention Sensitivity Analysis eFigure 11. Simulation Results for Scenario 1, Base Case eFigure 12. Simulation Results for Scenario 2, Increased Harm Reduction eFigure 13. Simulation Results for Scenario 3, Improved Testing eFigure 14. Simulation Results for Scenario 4, Improved Treatment eFigure 15. Simulation Results for Scenario 5, Improved Testing and Treatment eFigure 16. Simulation Results for Scenario 6, Improved Testing, Treatment, and Harm Reduction eFigure 17. Sensitivity Analysis to Limit on Treatment Rate in Scenario 6 [file jamanetwopen-e2119092-s001.pdf]

Supplemental Online Content

Blake A, Smith JE. Modeling hepatitis C elimination among people who inject drugs in New Hampshire. *JAMA Netw Open.* 2021;4(8):e2119092. doi:10.1001/jamanetworkopen.2021.19092

eAppendix. Supplementary Methods and Results

eReferences.

eFigure 1. Drug Overdose Rates

eFigure 2. Injector Duration Submodel Structure

eFigure 3. MAT/SSP Enrollment Submodel Structure

eFigure 4. Model Forecasts for Scenario 1, Base Case

eFigure 5. Model Forecasts for Scenario 2, Improved Harm Reduction

eFigure 6. Model Forecasts for Scenario 3, Improved Testing

eFigure 7. Model Forecasts for Scenario 4, Improved Treatment

eFigure 8. Model Forecasts for Scenario 5, Improved Testing and Treatment

eFigure 9. Model Forecasts for Scenario 6, Improved Testing, Treatment and Harm Reduction

eFigure 10. Policy Intervention Sensitivity Analysis

eFigure 11. Simulation Results for Scenario 1, Base Case

eFigure 12. Simulation Results for Scenario 2, Increased Harm Reduction

eFigure 13. Simulation Results for Scenario 3, Improved Testing

eFigure 14. Simulation Results for Scenario 4, Improved Treatment

eFigure 15. Simulation Results for Scenario 5, Improved Testing and Treatment

eFigure 16. Simulation Results for Scenario 6, Improved Testing, Treatment, and Harm Reduction

eFigure 17. Sensitivity Analysis to Limit on Treatment Rate in Scenario 6

This supplemental material has been provided by the authors to give readers additional information about their work.

© 2021 Blake A et al. *JAMA Network Open.*

eAppendix. Supplementary Methods and Results

New Hampshire Context

New Hampshire (NH) provides an interesting setting for studying HCV elimination among PWID in the United States (US) because of its historically low rates of HCV testing and treatment and its high rates of injection drug use during the opioid crisis. Though the rate of injection drug use in NH is high compared to most other states, the lack of infrastructure for testing, treatment, and harm-reduction services appears to be common throughout the US. This section provides some background on the current state of injection drug use, HCV prevalence, HCV testing, HCV treatment, and harm-reduction efforts in NH and, when possible, how NH compares to other states.

Injection Drug Use in New Hampshire

Rates of injection drug use rose substantially in NH during the opioid crisis, with a clear increase in past-year heroin use from 2013-2018 observed in the National Surveys on Drug Use and Health.[1] This increased use of injection drug use is reflected in a rapid increase in overdose deaths during this period (eFigures 1a and b). Heroin and fentanyl are now responsible for the majority of drug overdoses in the state[2] (eFigure 1b). The majority of overdose deaths, ED visits, and EMS naloxone occur among individuals age 20-49, mirroring national trends in HCV incidence attributable to PWID.[3] Rising rates of complications related to injection drug use – such as infective endocarditis or outbreaks of HIV similar to that seen in Scott County, Indiana – have also been noted.[4,5]

NH has consistently been among states with the highest overdose mortality since 2014.[6] A 2017 "hotspot" report prepared by the National Drug Early Warning System studied this issue in some detail, identifying reasons for the rapid growth in overdoses from 2010-2016. Reasons identified include high rates of substance abuse, high historical rates of opioid prescribing, and poor access to substance abuse treatment and harm reduction programs (more below).[7]

HCV Prevalence, Testing, and Treatment:

Recent work by Romo et al. suggests HCV prevalence among PWID in NH is high though consistent with previous studies in the US.[8,9] HCV was not a reportable disease in NH until November 3, 2016, and there is no state-reported HCV prevalence data before then. Moreover, little testing has been done in potentially high-contact settings for PWID such as syringe service programs (SSPs) or medication-assisted treatment (MAT) programs.[10,11] Medicaid restrictions on treatment were strict in NH before 2016: patients were required to have F3 or greater liver disease, six months of sobriety and prescribers had to be a specialist or a physician with continuing medical education in HCV treatment.[12] These restrictions were relaxed in 2016, but prescribers must still document addiction treatment and periodic screens for alcohol use.[12] Such restrictions have limited HCV testing and treatment in NH and impeded HCV elimination efforts.[8,13]

Though comprehensive data on PWID HCV testing and treatment in the US is not available, many studies suggest that insufficient rates of testing and treatment are common throughout the US.[8,14–16] Recent published editorials from HCV experts describe a paucity of testing and treatment in the US for PWID,[17,18] though they do not comment on state-by-state specifics. Taken together, this suggests the low rates of testing and treatment in NH are common in other states as well.

Harm-reduction

There were no legally operating SSPs in NH before 2017.[19] Since SSPs were legalized in NH in 2017, there has been continued growth in the number of SSPs and the number of total syringes distributed in NH.[11] Previous studies demonstrate significant heterogeneity state-by-state in SSP coverage, leaving many PWID with insufficient access to clean needles.[20,21] Access to SSPs has grown during the opioid crisis – in NH and across the country – but legal barriers remain in many places. Overall, the evidence suggests that poor access to SSPs is common in the US.[20]

As in many states, the capacity for treatment in MAT was far below the estimated prevalence of opioid use disorder at the beginning of the opioid crisis.[22] The 2017 "hotspot" report described above notes that NH has historically had low funding for public health, low rates of substance abuse treatment, and few buprenorphine-waivered prescribers.[7] In response to the opioid crisis, state leaders have dedicated millions of dollars to expanding treatment options in NH. Data from the National Survey of Substance Abuse Treatment Services show substantial growth in the number of MAT facilities and MAT participation in NH and across the country from 2013-2019.[10,23]

New Hampshire Surveillance Data

Precise data on PWID are lacking both in NH and across the US. Our analysis relies on state-specific data on the HCV burden among HCV collected from a variety of sources cited throughout the main paper and supplement. In this section, we provide further detail on these state-specific sources and their methodologies. We also used data from the National Surveys on Drug Use and Health (NSDUH) and National Survey of Substance Abuse Treatment Services (N-SSAT), both available at the Substance Abuse and Mental Health Services Administration (SAMHSA) website.[24]

Reportable Communicable Diseases in New Hampshire

As mandated by state law, the NH Department of Health and Human Services regularly collects and publishes case counts of select communicable diseases in the state.[25] Acute and chronic HCV incidence has been included in this report since November 3, 2016. Chronic HCV case counts in this report represent new diagnoses of chronic HCV. Data are also collected on whether HCV cases (acute and chronic) are related to injection drug use. HCV incidence stratified by injection drug use status is available from the state upon request. Stratified data was used for model validation of HCV incidence as described in the Validation section below (data as of 2/3/2021).[26] The communicable diseases report series is based on information reported to the Department of Health and Human Services and may represent an underestimate of the true absolute number and incident rates of cases in the state.

Drug Injection Surveillance and Care Enhancement for Rural Northern New England (DISCERNNE) Study

The DISCERNNE study is a multisite, multidisciplinary study conducting epidemiological and policy assessment of opioid-related outcomes, prevention and care services, and laws in eleven contiguous counties in rural MA, VT, and NH. Data was collected using both quantitative and qualitative methods including HCV testing, quantitative and social network survey, and PWID interviews on drug use and treatment histories. Participants were recruited using respondent-driven sampling and included 199 NH PWID. The details of this study have been published previously and provide useful context on the opioid crisis in the rural northeast.[8,27]

New Hampshire Drug Monitoring Initiative (DMI)

The DMI's stated purpose is to provide awareness and combat drug distribution and abuse. The DMI obtains data from various sources (including Public Health, Law Enforcement, and EMS) and provides monthly reports for stakeholders as well as situational awareness releases as needed. These data include overdose deaths, ED visits, EMS naloxone administration, and treatment admissions stratified by age and location.[28]

© 2021 Blake A et al. *JAMA Network Open.*

Model Details

We built a compartmental, deterministic, dynamic HCV transmission model whose compartments represent the number of people in a particular category. While most of the model components are based on similar models described previously,[29–31] the testing and treatment model is novel. All calculations were performed using MATLAB R2019b.

The number of people in a given compartment is denoted $Y_{i,j,k}^{n,m,d}$ where the superscripts and subscripts represent the following dimensions of the model:

- n = 1, 2, 3, 4 for recent PWID, non-recent PWID, long-term PWID (collectively referred to as active-PWID), and inactive-PWID, respectively.
- m = 0, 1 for comorbid opioid and stimulant use (1) or not (0).
- d = 1, …, 12 for the states in the testing and treatment model (see Figure 1B). d=1, …, 5 are the susceptible states (negative < 6 months ago, …, negative > 24 months ago). d=6, …, 10 are the corresponding infected undiagnosed states; d=11 is linked to care and d=12 is lost to follow up.
- i = 0, 1 enrolled in MAT (1) or not (0).
- j = 0, 1 enrolled in SSP (1) or not (0).
- k = 1, …, 9 for the stages of liver disease in Figure 1A: F0, F1, F2, F3, compensated cirrhosis (F4), decompensated cirrhosis, hepatocellular carcinoma, liver transplant and post liver transplant, respectively.

Thus, there are a total of 4 × 2 × 12 × 2 × 2 × 9 = 3,456 compartments in the model.

PWID transition through model compartments via a system of differential equations with derivatives given by

$$\frac{d\,Y_{i,j,k}^{n,m,d}}{dt} = IN_{i,j,k}^{n,m,d} + ID_{i,j,k}^{n,m,d} + CS_{i,j,k}^{n,m,d} + IT_{i,j,k}^{n,m,d} + DS_{i,j,k}^{n,m,d} + FOI_{i,j,k}^{n,m,d} + T_{i,j,k}^{n,m,d}\,,$$

where each term above is described below.

Inflow of PWID (IN)

PWID enter the model at rate θ. They are assumed to be initiating injection drug use for the first time (n=1); they are known to not have HCV (d=1), do not have liver disease (k=1), and are not enrolled in MAT or SSP (i=0, j=0). A fraction ϕ of these entering PWID are comorbid stimulant users (m=1); the remainder are not (m=0). Thus

$$IN_{0,0,1}^{1,0,1} = \theta\,(1-\phi)$$
$$IN_{0,0,1}^{1,1,1} = \theta\,\phi$$

All other $IN_{i,j,k}^{n,m,d}$ terms are zero.

The inflow rate θ varies over time, with one inflow rate before 2013, another for the time of the opioid epidemic in 2013-2018, and afrer 2018. See the Model Calibration section below for more details. We assume ϕ is 16%.[28] In the Monte Carlo simulation, ϕ is drawn from a uniform distribution ranging from 10%-22%.

Injector Duration Progression (ID)

eFigure 2 depicts the structure of the injector duration submodel. PWID progress through PWID duration categories at rates τ_n unless they become abstinent; the cessation rate is ω_n. Abstinent PWID relapse at rate μ. PWID exit the model with mortality rates ν_a and ν_i for active and inactive injectors; we assume higher mortality rates for active injectors The injector duration (ID) terms in the model are thus:

$$\begin{pmatrix} ID_{i,j,k}^{1,m,d} \\ ID_{i,j,k}^{2,m,d} \\ ID_{i,j,k}^{3,m,d} \\ ID_{i,j,k}^{4,m,d} \end{pmatrix} = \begin{pmatrix} -\tau_1 - \nu_a - \omega & 0 & 0 & \mu \\ \tau_1 & -\tau_2 - \nu_a - \omega & 0 & 0 \\ 0 & \tau_2 & -\nu_a - \omega & 0 \\ \omega & \omega & \omega & -\mu - \nu_i \end{pmatrix} \begin{pmatrix} Y_{i,j,k}^{1,m,d} \\ Y_{i,j,k}^{2,m,d} \\ Y_{i,j,k}^{3,m,d} \\ Y_{i,j,k}^{4,m,d} \end{pmatrix}$$

The assumed parameter values are as follows:

Parameter	Description	Base Case Value	Distribution for Simulation	Source
τ_1	Aging rate	0.200	-	5 years in category
τ_2	Aging rate	0.333	-	3 years in category
ν_a	Mortality Active	0.0264	Uniform (0.0125,0.0402)	North America average in Mather et al. 2013[32]
ν_i	Mortality Inactive	0.00154	-	Average death rate for 20-50 yr olds in Arias et al. 2019[33]
ω	Cessation rate	0.17	Beta(936,4617)	Durham et al. 2016[34]
μ	Relapse rate	0.085	Uniform(0.07,0.10)	Heroin users in Noysk et al. 2014[35]

Comorbid Stimulant Use (CS)

The model recognizes comorbid use of stimulants such as cocaine or amphetamines as leading to higher risk for HCV infection.[29] PWID go through periods of stimulant use, entering and exiting at rates σ and ζ respectively. Thus the comorbid stimulant (CS) terms in the model are:

$$\begin{pmatrix} CS_{i,j,k}^{n,0,d} \\ CS_{i,j,k}^{n,1,d} \end{pmatrix} = \begin{pmatrix} -\sigma & \zeta \\ \sigma & -\zeta \end{pmatrix} \begin{pmatrix} Y_{i,j,k}^{n,0,d} \\ Y_{i,j,k}^{n,1,d} \end{pmatrix}$$

The entering rate σ is selected to match a specified steady-state fraction f_2 in this high-risk category.

The assumed parameter values are as follows:

Parameter	Description	Base Case Value	Distribution for Simulation	Reference
ζ	Leaving Rate	0.805	Uniform(0.66,0.95)	Adapted from episode durations in Nosyk et al. 2014[35]
f_2	Fraction High Risk	17%	Uniform(10%,24%)	Adapted from DMI[28]

MAT/SSP Enrollment (IT)

eFigure 3 depicts the structure of the MAT/SSP submodel. We assume entering or leaving one form of harm reduction is independent of the other. Active PWID enter MAT and SSP at rates β and η, respectively. PWID exit MAT and SSP at rates γ and κ respectively. Thus for active injectors (n=1, 2, 3), the intervention terms (IT) in the model are

$$\begin{pmatrix} IT_{0,0,k}^{n,m,d} \\ IT_{1,0,k}^{n,m,d} \\ IT_{0,1,k}^{n,m,d} \\ IT_{1,1,k}^{n,m,d} \end{pmatrix} = \begin{pmatrix} -\eta-\beta & \gamma & \kappa & 0 \\ \beta & -\eta-\gamma & 0 & \kappa \\ \eta & 0 & -\kappa-\beta & \gamma \\ 0 & \eta & \beta & -\gamma-\kappa \end{pmatrix} \begin{pmatrix} Y_{0,0,k}^{n,m,d} \\ Y_{1,0,k}^{n,m,d} \\ Y_{0,1,k}^{n,m,d} \\ Y_{1,1,k}^{n,m,d} \end{pmatrix}.$$

Inactive injectors (*n*=4) do not enter SSP and exit immediately upon becoming abstinent. The IT terms for inactive injectors are as above but with $\eta = 0$ and $\kappa \approx \infty$.

The entering rates β and η varied across different policy scenarios and were chosen to match steady-state enrollment targets for the given scenario. The assumed leaving rates were as follows:

Parameter	Description	Base Case Value	Reference
γ	MAT Leaving Rate	1.50	Fraser et al.[29]
κ	SSP Leaving Rate	0.54	Fraser et al.[29]

The scenario-specific steady-state enrollment targets were set as follows:

- Pre-2013:
  MAT Enrollment Target = 7.5%
  SSP Enrollment Target = 0%
  The pre-2013 MAT enrollment target is estimated from an observed 1.53x increase in MAT participation from 2013-2020.[10,23] We assume continued growth in MAT coverage in the base case such that the 2022 target is twice the pre-2013 target. There were no SSPs in NH before 2013 and this is assumed to be 0%.

- Base case harm prevention scenarios (Scenarios 1, 3, 4, 5), also 2013-2022:
  MAT Enrollment Target = 15% [36]
  SSP Enrollment Target = 15% [11]
  Base case harm prevention targets are based on reported opioid use disorder treatment rates nationally (19.7%), adjusted for below average rates of buprenorphine-waivered providers and rates of admissions for substance use in NH.[7,36] Base case SSP coverage target is based on state-reported data for the number of clean needles distributed.[11]

- Increased harm prevention scenarios (Scenarios 2, 6):
  MAT Enrollment Target = 35%
  SSP Enrollment Target = 50%
  Increased harm prevention scenario targets were selected based on prior work by Fraser et al. who assume targets of 50% for both MAT and SSP; we assume a more modest scale up in MAT participation.[37]

- Intermediate harm prevention scenario (Policy Sensitivity Analysis of eFigure 9):
  MAT Enrollment Target = 25%
  SSP Enrollment Target = 32.5%

Infection (FOI)

Acquiring a chronic HCV Infection in the model corresponds to uninfected people (those with *d*=1,…, 5) moving from compartment $Y_{i,j,k}^{n,m,d}$ to the corresponding infected compartment $Y_{i,j,k}^{n,m,d+5}$. The force of infection (FOI) terms in the model capture this process. For *d*=1,…, 5, the FOI terms are of the form

$$FOI_{i,j,k}^{n,m,d} = -\pi(1-\delta)\,\Upsilon\,\lambda_{i,j}^{n,m}\,Y_{i,j,k}^{n,m,d}$$
$$FOI_{i,j,k}^{n,m,d+5} = -FOI_{i,j,k}^{n,m,d} \quad ,$$

© 2021 Blake A et al. *JAMA Network Open.*

representing the flow out of the uninfected states and into the corresponding infected states. All other FOI terms are zero. Here

- π represents the intrinsic risk of HCV acquisition with injection,
- δ is the fraction who spontaneously clear an acute infection; hence fraction $(1-\delta)$ become chronically infected,
- $\lambda_{i,j}^{n,m}$ is a weight representing additional risk or protection associated with specific categories

$$\lambda_{i,j}^{n,m} = X_n \, \Xi_m \, \Gamma_i \, \Pi_j$$

  where

  - X_n is the relative weight by injector category; here $X_3 = 1$ because weights are relative to long-term injectors and $X_4 = 0$ because inactive PWID do not face transmission risk.
  - Ξ_m, Γ_i, and Π_j are relative adjustments for comorbid stimulant use, enrollment in MAT and enrollment in SSP, respectively. $\Xi_0 = \Gamma_0 = \Pi_0 = 1$, defining the base of the relative adjustment.

- Υ represents a risk-weighted fraction of total PWID that are infectious and is given as

$$\Upsilon = \frac{\sum_{d=6}^{12} \quad \sum_{i,j,k,m,n} \lambda_{i,j}^{n,m} \, Y_{i,j,k}^{n,m,d}}{\sum_{d=1}^{12} \quad \sum_{i,j,k,m,n} \lambda_{i,j}^{n,m} \, Y_{i,j,k}^{n,m,d}}$$

  Here the numerator is the risk-weighted total number of infected PWID (*d* = 6 … 12) and the denominator is the risk-weighted total number of people, whether they are infected or not. Note that the risk-weighting ensure that only active injectors are included in these totals, since $\lambda_{i,j}^{n,m} = 0$ for inactive injectors (*n*=4).

The intuition behind this model is that PWID interact (e.g., by sharing needles) with other PWID with the risk-weighting terms reflecting the frequency of interaction and likelihood for the interaction to result in transmission of HCV.

The intrinsic risk π is assumed to be different before and after 2013 to capture the change in PWID behavior with the onset of the opioid crisis. See Model Calibration below for more information.

The assumed parameter values are as follows.

Parameter	Description	Base Case Value	Distribution for Simulation*	Source
X_1	Risk factor for recent injectors	2.60	LN(2.65,0.58)	Adapted from Roy et al. 2009 [38]
X_2	Risk factor for non-recent injectors	1.48	LN(1.48,0.32)	Adapted from Roy et al. 2009 [38]
Ξ_1	Risk factor for comorbid stimulant use	2.13	LN(2.18,1.03)	Ward et al.[29]
Γ_1	Risk factor for enrollment in MAT	0.50	LN(0.50,0.03)	Fraser et al.[37,39]
Π_1	Risk factor for enrollment in SSP	0.44	LN(0.46,0.07)	Fraser et al.[37,39]
δ	Spontaneous clearance rate	25.5%	Uniform(0.22,0.29)	Fraser et al.[37]

Here LN(μ, σ) denotes a log-normal distribution with mean μ and standard deviation σ.

Liver Disease Progression (DS)

Figure 1A in the main paper depicts the structure of the liver disease progression submodel. The disease progression rates depend on HCV infection status. For infected people (*d*=6, …, 12), the disease progression (DS) terms in the model are

$$\begin{pmatrix} DS_{i,j,1}^{n,m,d} \\ DS_{i,j,2}^{n,m,d} \\ DS_{i,j,3}^{n,m,d} \\ DS_{i,j,4}^{n,m,d} \\ DS_{i,j,5}^{n,m,d} \\ DS_{i,j,6}^{n,m,d} \\ DS_{i,j,7}^{n,m,d} \\ DS_{i,j,8}^{n,m,d} \\ DS_{i,j,9}^{n,m,d} \end{pmatrix} =$$

$$\begin{pmatrix} -\rho_1 & 0 & 0 & 0 & 0 & 0 & 0 & 0 & 0 \\ \rho_1 & -\rho_2 & 0 & 0 & 0 & 0 & 0 & 0 & 0 \\ 0 & \rho_2 & -\rho_3 & 0 & 0 & 0 & 0 & 0 & 0 \\ 0 & 0 & \rho_3 & -\rho_4 & 0 & 0 & 0 & 0 & 0 \\ 0 & 0 & 0 & \rho_4 & -\rho_5-\rho_{6a} & 0 & 0 & 0 & 0 \\ 0 & 0 & 0 & 0 & \rho_5 & -\rho_{6b}-\rho_{7a}-d_6 & 0 & 0 & 0 \\ 0 & 0 & 0 & 0 & \rho_{6a} & \rho_{6b} & -\rho_{7b}-d_7 & 0 & 0 \\ 0 & 0 & 0 & 0 & 0 & \rho_{7a} & \rho_{7b} & -\rho_8-d_8 & 0 \\ 0 & 0 & 0 & 0 & 0 & 0 & 0 & \rho_8 & -d_9 \end{pmatrix} \begin{pmatrix} Y_{i,j,1}^{n,m,d} \\ Y_{i,j,2}^{n,m,d} \\ Y_{i,j,3}^{n,m,d} \\ Y_{i,j,4}^{n,m} \\ Y_{i,j,5}^{n,m} \\ Y_{i,j,6}^{n,m,d} \\ Y_{i,j,7}^{n,m} \\ Y_{i,j,8}^{n,m,d} \\ Y_{i,j,9}^{n,m,d} \end{pmatrix}.$$

Here the ρ_k are disease progression rates and the d_k are increased mortality rates associated with advanced stages of liver disease.

Uninfected people (*d*=1, …, 5) do not progress through the first four liver disease states and have different rates of progression through the 5th and 6th stages of liver disease (e_5 and e_6 instead of ρ_5 and ρ_{6a}). For these people, the DS terms in the model are

$$\begin{pmatrix} DS_{i,j,1}^{n,m,d} \\ DS_{i,j,2}^{n,m,d} \\ DS_{i,j,3}^{n,m,d} \\ DS_{i,j,4}^{n,m,d} \\ DS_{i,j,5}^{n,m,d} \\ DS_{i,j,6}^{n,m,d} \\ DS_{i,j,7}^{n,m,d} \\ DS_{i,j,8}^{n,m,d} \\ DS_{i,j,9}^{n,m,d} \end{pmatrix} = \begin{pmatrix} 0 & 0 & 0 & 0 & 0 & 0 & 0 & 0 & 0 \\ 0 & 0 & 0 & 0 & 0 & 0 & 0 & 0 & 0 \\ 0 & 0 & 0 & 0 & 0 & 0 & 0 & 0 & 0 \\ 0 & 0 & 0 & 0 & 0 & 0 & 0 & 0 & 0 \\ 0 & 0 & 0 & 0 & -e_5-e_6 & 0 & 0 & 0 & 0 \\ 0 & 0 & 0 & 0 & e_5 & -\rho_{6a}-\rho_{7a}-d_6 & 0 & 0 & 0 \\ 0 & 0 & 0 & 0 & e_6 & \rho_{6a} & -\rho_{7b}-d_7 & 0 & 0 \\ 0 & 0 & 0 & 0 & 0 & \rho_{7a} & \rho_{7b} & -\rho_8-d_8 & 0 \\ 0 & 0 & 0 & 0 & 0 & 0 & 0 & \rho_8 & -d_9 \end{pmatrix} \begin{pmatrix} Y_{i,j,1}^{n,m,d} \\ Y_{i,j,2}^{n,m,d} \\ Y_{i,j,3}^{n,m,d} \\ Y_{i,j,4}^{n,m} \\ Y_{i,j,5}^{n,m} \\ Y_{i,j,6}^{n,m,d} \\ Y_{i,j,7}^{n,m} \\ Y_{i,j,8}^{n,m,d} \\ Y_{i,j,9}^{n,m,d} \end{pmatrix}.$$

Our disease progression rates are based on Kabiri et al. and are converted from the annual transition probabilities given there to transition rates as used here.[40] Given an annual transition probability p, the corresponding transition rate is $\rho = -\log(1-p)$. The exception to this is the liver transplant to post-liver transplant transition ρ_8 which is

© 2021 Blake A et al. *JAMA Network Open.*

modeled as a deterministic process by Kaberi taking one year, by definition, since post-liver transplant is defined to be one year post-transplant. Thus we take $\rho_8 = 1$.

Parameter	Description	Base Case Value	Distribution for Simulation: Uniform with Lower Limit	Upper Limit
ρ_1	F0 -> F1	0.1244	0.1098	0.1393
ρ_2	F1 -> F2	0.0888	0.0780	0.1009
ρ_3	F2 -> F3	0.1278	0.1154	0.1427
ρ_4	F3 -> Compensated cirrhosis (F4)	0.1233	0.1098	0.1381
ρ_5	F4 -> Decompensated cirrhosis (DC)	0.0294	0.0101	0.0398
ρ_{6a}	F4 -> Hepatocellular carcinoma (HCC)	0.0141	0.0101	0.0823
ρ_{6b}	DC -> HCC	0.0704	0.0305	0.0866
ρ_{7a}	DC -> Liver transplant	0.0233	0.0101	0.0640
ρ_{7b}	HCC -> Liver transplant	0.0408	0.0000	0.1508
ρ_8	Liver transplant -> post liver transplant	1.0000	1.0000	1.0000
d_6	DCC death rate	0.1188	0.0672	0.2107
d_7	HCC death rate	0.5569	0.4005	1.9661
d_8	Liver transplant death rate	0.1233	0.0619	0.5447
d_9	Post liver transplant death rate	0.0450	0.0243	0.1165
e_5	F4 -> DC when not infected	0.0080	0.0020	0.0367
e_6	F4 -> HCC following when not infected	0.0050	0.0020	0.0131

Testing and Treatment (TT)

The structure of the testing and treatment submodel is depicted Figure 1B in main paper.

PWID (both susceptible and infected) who are not tested "age" from one testing category to the next (e.g., from < 6 months to 6-12 months since the last negative test result) until they reach the final category (> 24 months) where they remain until tested. The aging rate ν = 2.0 reflects the fact the time intervals are 6 months (=1/2 year) in length. PWID who are not infected and are tested revert to the "< 6 months" (d=1) since last negative category. Those PWID who are infected and are tested become linked to care (d=11). The testing rates $\Delta^p_{i,j,k}$ depend on enrollment in MAT/SSP (the i and j indices), the liver disease state (k index), and the time since last tested negative (captured through p index). The specific rate assumptions will vary by scenarios.

PWID who are linked to care exit via treatment at rate ξ^1_{kn} or are lost to follow up at rate ξ^2_{kn}, with the rates potentially depending on the liver disease state (the k index) and the injector status (the n index), to capture restrictions related to disease or sobriety in some scenarios.

PWID who are lost to follow up are reengaged at rate $\Delta^1_{i,j,k}$, corresponding to the testing rate for the given category for a person who tested negatively (p=1) in the last 6 months; the assumption is that people get reengaged with care through a process that is similar to the process of becoming linked to care. PWID receiving treatment achieve a sustained viral response (SVR) at rate α.

Combining the testing and treatment processes, the testing and treatment (TT) terms in the model are

$$
\begin{pmatrix} T_{i,j,k}^{n,m,1} \\ T_{i,j,k}^{n,m,2} \\ T_{i,j,k}^{n,m,3} \\ T_{i,j,k}^{n,m,4} \\ T_{i,j,k}^{n,m,5} \\ T_{i,j,k}^{n,m,6} \\ T_{i,j,k}^{n,m,7} \\ T_{i,j,k}^{n,m,8} \\ T_{i,j,k}^{n,m,9} \\ T_{i,j,k}^{n,m,10} \\ T_{i,j,k}^{n,m,11} \\ T_{i,j,k}^{n,m,12} \end{pmatrix} =
$$

$$
\begin{pmatrix}
-\nu & \Delta_{i,j,k}^{2} & \Delta_{i,j,k}^{3} & \Delta_{i,j,k}^{4} & \Delta_{i,j,k}^{5} & 0 & 0 & 0 & 0 & 0 & \alpha\xi_{kn}^{1} & 0 \\
\nu & -\nu-\Delta_{i,j,k}^{2} & 0 & 0 & 0 & 0 & 0 & 0 & 0 & 0 & 0 & 0 \\
0 & \nu & -\nu-\Delta_{i,j,k}^{3} & 0 & 0 & 0 & 0 & 0 & 0 & 0 & 0 & 0 \\
0 & 0 & \nu & -\nu-\Delta_{i,j,k}^{4} & 0 & 0 & 0 & 0 & 0 & 0 & 0 & 0 \\
0 & 0 & 0 & \nu & -\Delta_{i,j,k}^{5} & 0 & 0 & 0 & 0 & 0 & 0 & 0 \\
0 & 0 & 0 & 0 & 0 & -\nu-\Delta_{i,j,k}^{1} & 0 & 0 & 0 & 0 & 0 & 0 \\
0 & 0 & 0 & 0 & 0 & \nu & -\nu-\Delta_{i,j,k}^{2} & 0 & 0 & 0 & 0 & 0 \\
0 & 0 & 0 & 0 & 0 & 0 & \nu & -\nu-\Delta_{i,j,k}^{3} & 0 & 0 & 0 & 0 \\
0 & 0 & 0 & 0 & 0 & 0 & 0 & \nu & -\nu-\Delta_{i,j,k}^{4} & 0 & 0 & 0 \\
0 & 0 & 0 & 0 & 0 & 0 & 0 & 0 & \nu & -\Delta_{i,j,k}^{5} & 0 & 0 \\
0 & 0 & 0 & 0 & 0 & \Delta_{i,j,k}^{1} & \Delta_{i,j,k}^{2} & \Delta_{i,j,k}^{3} & \Delta_{i,j,k}^{4} & \Delta_{i,j,k}^{5} & -\alpha\xi_{kn}^{1}-\xi_{kn}^{2} & \Delta_{i,j,k}^{1} \\
0 & 0 & 0 & 0 & 0 & 0 & 0 & 0 & 0 & 0 & \xi_{kn}^{2} & -\Delta_{i,j,k_3}^{1}
\end{pmatrix}
\begin{pmatrix} Y_{i,j,k}^{n,m,1} \\ Y_{i,j,k}^{n,m,2} \\ Y_{i,j,k}^{n,m,3} \\ Y_{i,j,k}^{n,m,4} \\ Y_{i,j,k}^{n,m,5} \\ Y_{i,j,k}^{n,m,6} \\ Y_{i,j,k}^{n,m,7} \\ Y_{i,j,k}^{n,m,8} \\ Y_{i,j,k}^{n,m,9} \\ Y_{i,j,k}^{n,m,10} \\ Y_{i,j,k}^{n,m,11} \\ Y_{i,j,k}^{n,m,12} \end{pmatrix}
$$

Testing Parameters. The assumed treatment parameters vary by timeframe and across scenarios. In all cases, the testing rates $\Delta_{i,j,k}^{p}$ were decomposed into factor-specific terms the form

$$\Delta_{i,j,k}^{p} = b \times d_k \times v_{ij}^{p}$$

where

- b is a base testing rate,
- d_k is a multiplier that captures the idea that people who are asymptomatic for liver disease (i.e., in METAVIR stages F0, F1, F2, F3 corresponding k=1,2,3, 4) are less likely to be tested for HCV and those with more advanced liver disease are more likely to be tested.
- v_{ij}^{p} is a multiplier that captures dependence on how long it has been since the person was last tested (p) and whether they are enrolled in MAT/SSP (i,j).

The assumed values vary by scenario as follows:

- Base Case Testing scenarios (Scenarios 1, 2, and 4), Pre 2013, and 2013-2022:

  $b = 2.0$
  $[d_1 \ldots d_9] = [0.025\ \ 0.025\ \ 0.025\ \ 0.025\ \ 0.1\ \ 0.4\ \ 0.4\ 1.0\ 1.0]$
  $v_{ij}^{p} = 1$ for all p, i, j

  These parameters imply that somebody who is asymptomatic (k=1,…, 4) would be tested at rate 2.0 × 0.025 × 1 = 0.05, i.e., at a rate of once per 20 years. The disease states d_k adjustments are from Heffernan et al.[2] and the base rate b was chosen to match diagnosis rates assumed in earlier studies focused on PWID

in the US.[20]

- For the Improved Testing scenarios (Scenarios 3, 5, and 6):

b = 2.0
$[d_1 \ldots d_9]$ = [0.25 0.25 0.25 0.25 0.4 0.4 0.4 1.0 1.0]
$[v_{00}^1 \ldots v_{00}^5]$ = [1 1 1 1 1] (not in MAT or SSP)
$[v_{ij}^1 \ldots v_{ij}^5]$ = [1 1 4 4 4] if i = 1 or j= 1 (if in MAT or SSP)

These parameters imply that somebody who is asymptomatic (k=1,…, 4) would be tested at rate 2.0 × 0. 25 × 1 = 0.5, i.e., at a rate of once per 2 years if not in MAT/SSP, independent of when they had last been tested. If in MAT or SSP, the same person would have no chance of being tested if they had tested negative in the last year and would be tested at rate 2.0 × 0. 25 × 4 = 2, i.e., once per 6 months, if they had not been tested in the last year.

- In the four community testing rates scenarios considered in the Policy Sensitivity Analysis of eFigure 9, we took b = 2.0 and considered

1. $[d_1 \ldots d_9]$ = [0.025 0.025 0.025 0.025 0.1 0.4 0.4 1.0 1.0]
2. $[d_1 \ldots d_9]$ = [0.05 0.05 0.05 0.05 0.1 0.4 0.4 1.0 1.0]
3. $[d_1 \ldots d_9]$ = [0.10 0.10 0.10 0.10 0.1 0.4 0.4 1.0 1.0]
4. $[d_1 \ldots d_9]$ = [0.25 0.25 0.25 0.25 0.5 0.4 0.4 1.0 1.0]

With these assumptions, the asymptomatic testing rate varies from that of the Base Case Testing scenario to that of the Improved Testing scenario. The v_{ij}^p terms in these scenarios are as in the Base Case Testing scenario when there is community testing only and as in the Increased Testing scenario when there was annual testing at the MAT and SSP.

Treatment Parameters. The assumed treatment parameters vary by timeframe and across scenarios. In the base case scenario, the treatment rate ξ^1 reflects the time required to successfully complete treatment. The loss rates ξ^2 were selected to fit published data on attrition through the HCV care cascade.[8] Given treatment and loss rates ξ^1 and ξ^2, a fraction $f = \xi^1/(\xi^1 + \xi^2)$ of those linked to care will actually be treated. We chose these fractions f_{kn} and solved for the loss-to-follow-up rate $\xi^2 = (1 - f)\, \xi^1/f$. (We suppress the dependence of the treatment rates and fractions on the liver disease state k and injector status n in this discussion.)

The assumed values vary by scenario as follows:

- Before 2013: (Before directing-acting antivirals)
  Treatment rate: ξ^1= 0.25
  Fraction treated:
  f = 0.68% for active injectors
  f = 2.5% for inactive injectors
  SVR Rate: α = 40%
  No treatment with advanced liver disease ($k \leq 6$)

  The treatment fractions were based on PWID treatment rates in Durham et al.[34]

- Base case Treatment scenarios (Policy Scenarios 1, 2, and 3) and 2013-2022:
  Treatment rate: ξ^1= 1.00
  Loss rate: f= 20%
  SVR Rate: α = 95%
  No treatment with advanced liver disease ($k \leq 6$)

  The 20% treatment fraction was estimated from Romo et al.'s HCV PWID care cascade.[8]

- Improved Treatment scenarios (Policy Scenarios 4, 5, and 6):

  Treatment rate: $\xi^1 = 4.0$

  Loss rate: $f = 100\%$

  SVR Rate: $\alpha = 95\%$

  No treatment with advanced liver disease ($k \leq 6$)

- Intermediate Treatment scenario for Policy Sensitivity Analysis of eFigure 9:

  Treatment rate: $\xi^1 = 2.0$

  Loss rate: $f = 60\%$

  SVR Rate: $\alpha = 95\%$

  No treatment with advanced liver disease ($k \leq 6$)

© 2021 Blake A et al. *JAMA Network Open.*

Model Calibration

To capture the effects of the opioid epidemic, the time horizon was divided into three time periods – before 2013, 2013-2018, and after 2018 – with the injector inflow rate θ varying across these time frames. The infectivity rate π is different before and after 2013.

In the deterministic models, we assumed:

- Before 2013, the population is assumed to be in steady-state and θ and π were selected to give a prevalence of 40% among all PWID[18] and a population of 4000 (=8000/2) active injectors in 2013. The prevalence estimate represents a US average for PWID RNA prevalence, as no NH-specific prevalence data is available in that time period. The population estimate is based on an estimate of 8000 active injectors in 2018 (see below) and a two-fold increase during the opioid crisis.[1,37,41] In the base case, this calibration process results in $\theta \approx 172$ (i.e., 172 new injectors entering per year) and $\pi \approx 0.069$.

- From 2013 to 2022, the inflow rates θ before and after 2018 were selected to match a population of 8000 active injectors in 2018 and a 10% reduction of active injectors in 2022, that is, 7200 active injectors in 2022. We chose 8000 active injectors based on NSDUH estimates of past-year heroin, cocaine, and meth use,[1] and adjusted these estimates taking into account rates of injection use for each drug.[42–44] This method produced a peak population size of between 4917-13741 active injectors. NSDUH past-year heroin use and NH DMI overdose mortality data suggest peak use occurred between 2016 (NSDUH) and 2018 (NH DMI; see eFigure 1b).[1,2] Because NSDUH data may not capture some changes in substance use behavior (such as a switch from heroin to fentanyl), we assumed a peak population in 2018. The assumed reduction in active injectors is based on declining rates of reported past-year heroin use and drug overdose in NH,[1,2,44] though we assume a more modest reduction than what is seen in this data, based on reports of rising substance use during the COVID-19 pandemic.[45] The infectivity rate π was selected to ensure a prevalence of 45% among all PWID in 2019 based on Romo et al. HCV seropositivity rates.[8] This calibration process results in $\pi \approx 0.298$ and $\theta \approx 1363$ before 2018 and 448 after 2018, reflecting an increase in both new injectors and infectivity associated with the opioid crisis.

- After 2022, π was assumed to be the same as after 2018 $\pi \approx 0.298$ and $\theta \approx 448$ as in the 2018-2022 period.

The parameters θ and π were selected using a nonlinear optimization routine that ensured that prevalence and population targets were met exactly. The before-2013 optimization is done first and the 2013 compartment values $Y_{i,j,k}^{n,m,d}$ become the initial state for a model focused on 2013-2022, whose parameters θ (before and after 2018) and π are chosen to match the 2019 prevalence and 2018 and 2022 population targets. The 2022 compartment values $Y_{i,j,k}^{n,m,d}$ then become the initial state for a model focused on 2022-2045.

To capture the uncertainty in these estimates, in the Monte Carlo simulations we assume:

- The 2018 number of active injectors is normally distributed with mean 8000 and standard deviation 1500.

- The 2013 number of active injectors is equal to the 2018 number of active injectors, divided by a random factor that is uniformly distributed from 1.5 to 2.5. This assumed relationship induces positive correlation between the 2013 and 2018 number of active injectors.

- The 2022 number of active injectors was assumed to be normally distributed with mean 90% of the 2018 number of active injectors and standard deviation 10% of this mean. By tying the mean and standard deviation here to the 2018 number of active injectors, we introduce positive correlation between the 2018 and 2022 numbers.

- The 2019 prevalence was taken to be normally distributed with mean 45% and standard deviation 5% and the 2013 prevalence as taken to be normally distributed with mean equal to 2019 prevalence less 5% and standard deviation 5%.

© 2021 Blake A et al. *JAMA Network Open.*

In each of the 500 trials of the simulation, the inflow rate θ and the intrinsic infection rates π for the three different periods were calibrated as described above to meet these randomly generated calibration targets, using the model parameters randomly generated in that trial.

© 2021 Blake A et al. *JAMA Network Open.*

Model Validation

To validate our model, we compared model outcomes to published estimates not used in parameter selection and the calibration process.[34] These outcomes included HCV prevalence, HCV incidence, new chronic HCV diagnoses, HCV treatments, and HCV mortality as shown in eTable 1. Because PWID-specific data is scarce, some of the validation measures are not exclusive to PWID; these include the HCV prevalence, treatments, and mortality. We include these observations as they may still be useful in assessing the validity of the model forecasts.

eTable 1: Validation Outcomes

Outcome	Years	Model Forecast	Validation Estimate	Source
HCV Prevalence[a]	2016	6144	7700	Rosenberg et al. 2018[46]
Acute HCV Infections	2017, 2018	2017: 914<br>2018: 911	2017: 391 (95% CI 256-1102);<br>2018: 454 (95% CI 297-1280)[b]	NH infectious disease surveillence[26]
Chronic HCV Diagnoses	2017-2019	211-272	193-261	NH infectious disease surveillance[26]
HCV Treatments[c]	2015-2017	101-108	288-326	Adapted from Chirikov et al 2018[47,48]
HCV Mortality[d]	2014-2018	54-56	57-68	CDC[3]

[a] Rosenberg et al. estimate HCV prevalence in NH from NHAENS data from 2013-2016. They estimate prevalence all HCV prevalence in NH, including but not limited to PWID.

[b] Validation estimate is calculated from NH infectious disease surveillance reporting as acute HCV cases attributable to injection drug use, corrected for underreporting (correction factor point estimate 16.8; 95% CI 11.0 – 47.4) and assuming a spontaneous clearance rate of 75%.[3,49]

[c]MappingHepC.com provides an estimate for all HCV treatments in NH, including but not limited to PWID. The published forecasts use a machine-learning algorithm to predict HCV treatments based on RNA testing results obtained from "two large national laboratory companies;" a decline in RNA viral loads in a sequence of tests is taken to be an indicator of successful treatment (Chirikov et al 2018). The lab data includes 3-digit zip codes for the ordering physician; this allows for state-specific estimates of treatments. The model forecast is calibrated using state-reported data.

[d]The CDC reports an estimate for all HCV mortality in NH, including but not limited to PWID.

© 2021 Blake A et al. *JAMA Network Open.*

Policy Sensitivity Analysis

In the six illustrative scenarios in the main paper, we considered various combinations of four dimensions of possible interventions – increased testing in the community (eg, primary care or emergency departments), scheduled testing in MAT/SSP, increased treatment uptake, and increased harm reduction coverage. To better understand the interactions among these dimensions, eFigure 10 shows the forecasted 2045 HCV prevalence and total HCV treatments associated with all possible combinations of interventions. In eFigure 10:

- The outer *x*-axis considers increasing access to/enrollment in harm reduction programs. The base case is as described above (15% of active PWID in SSP and 15% in MAT), the aggressive case is that in Scenario 2 above (50% in SSP and 35% in MAT) and the moderate case is halfway between (32.5% in SSP and 25% in MAT).

- The outer *y*-axis considers increased treatment uptake. The base case assumes 20% treated, the aggressive case is that considered in Scenarios 4, 5, and 6 (100% treated), and the moderate case is halfway between (60% treated). Detailed descriptions of the treatment model parameters in these three scenarios are in the Treatment Model discussion above.

- The *x*-axis of each bar chart shows the community testing rate. The red and blue bars show the 2045 prevalence with and without (respectively) annual testing at MAT/NSPs. In the scenarios without annual testing at MAT/NSPs, everybody is tested at the community rate. The community rate (1 per 20 years) is as in the base case and the highest (1 per 2 years) is as in Scenario 3. Detailed descriptions of these testing model parameters in these scenarios are in the Testing Model discussion above.

- The numbers above the prevalence bars show the total number of HCV treatments from 2022 to 2045 for each scenario.

In eFigure 10, we see that improvements in any of the four intervention dimensions were associated with a decreased 2045 prevalence. The lowest 2045 prevalence is achieved by pursuing all interventions aggressively, which corresponds to Scenario 6 in the main paper.

We also observe that the improvements associated with the interventions are synergistic. For example, the improvements associated with annual testing for those enrolled in harm reduction programs are indicated by the difference in heights for the blue and red bars in the graph. These differences increase (in relative terms) with increases in the treatment uptake, harm reduction, and testing rate. Thus the reduction in prevalence associated with an intervention (in this example, annual testing) is greater when pursued with other interventions.

Moreover, among the scenarios that achieve significant reductions in prevalence, the total number of treatments required decreases as the intensity of the intervention(s) increases. For example, the scenario with the maximum improvement on each dimension (Scenario 6) achieves near-zero prevalence with fewer treatments than any other combination of interventions associated with a 2045 prevalence to 30% or less. As discussed in the main paper, aggressive treatment early in the forecast period decreases the total number of treatments required over the remainder of the forecast period.

Monte Carlo Simulation Results

To test the robustness of our results, we conducted a Monte Carlo simulation analysis where we randomly generated 500 sets of model parameters according to the distributions described above. We generated model forecasts for the six scenarios described in the main paper for each set of model parameters. The results of the simulation are summarized in eFigures 11-16, with one figure for each of the six scenarios. The figures show the 10th, 50th, and 90th percentiles for key model forecasts from 2022 to 2045: prevalence, incidence (HCV infections and reinfections), HCV treatments and liver deaths.

The results of the simulation demonstrate the robustness of the conclusions from the deterministic analysis discussed in the main paper. Though there is considerable uncertainty in some outcomes, the two scenarios with improved testing and treatment (Scenarios 5 and 6) are the only ones that are consistently associated with a near-zero prevalence across this broad range of scenarios.

Scenario 6 (with increased harm reduction, testing, and treatment) is particularly robust in that model forecasts consistently show dramatic, rapid reductions in prevalence and incidence of HCV. The final prevalence ranges from 0.0% to 0.3% (10th and 90th percentiles) in the scenario. There is some uncertainty in the number of treatments required in this scenario, reflecting the uncertainty about the number infected in 2022 (which in turn affects the estimated intrinsic rate of infection). The testing and treatment resources in this scenario are adequate to handle this broad range of possible values. As in the deterministic analysis discussed in the paper, the number of treatments associated with Scenario 6 is less than Scenario 5.

In all scenarios, there is considerable uncertainty about the number of infections, treatments and liver deaths: this uncertainty reflects uncertainty about the number of PWID and the number infected in 2022, and for deaths, their state of liver disease, as well as uncertainty about the death rates and transplant rates. Scenarios 1-4 have consistently higher and sometimes increasing prevalences and rates of infections and deaths than Scenarios 5 and 6.

© 2021 Blake A et al. *JAMA Network Open.*

Treatment Rate Sensitivity Analysis

We also performed sensitivity analyses on Scenario 6 where we limit the number of annual treatments. The treatment rate in Scenario 6 is briefly over 3000 treatments per year in the first year of the intervention and it is natural to wonder what would happen if such a treatment rate were unattainable, for budget, supply, or other capacity limitations. To study this, we limit the treatment rate, considering limits of 500, 1000, 1500, 2000, 2500, 3000, and Infinity (i.e., no limit) treatments per year. In this analysis, when the demand for treatment exceeds the limit, treatments were prorated among those who would have been treated if there were no limits.

The results of this sensitivity analysis are summarized in eFigure 16. Here we see that with all of these limits, the prevalence is eventually near zero, but scenarios with lower limits reach this point later and were associated with more treatments in total. Placing a cap on treatments creates a bottleneck in the linked-to-care state as infected PWID are rapidly diagnosed, but cannot be treated as fast as they are identified. The larger limits considered (2000-3000 treatments per year) have little effect on the number of treatments required and the number of infections because these limits are reached only briefly. The lower treatment limits have increasingly severe negative impacts on all of the metrics considered.

© 2021 Blake A et al. *JAMA Network Open.*

eReferences

1. Substance Abuse & Mental Health Data Archive. Interactive NSDUH State Estimates. Published 2019. Accessed February 11, 2021. https://pdas.samhsa.gov/saes/state

2. *New Hampshire Drug Monitoring Initiative*. New Hampshire Department of Health and Human Services; 2021. Accessed April 2, 2021. https://www.dhhs.nh.gov/dcbcs/bdas/documents/dmi-feb-2021.pdf

3. Hepatitis C Surveillance in the United States, 2018. Centers for Disease Control and Prevention. Published November 18, 2020. Accessed January 4, 2021. https://www.cdc.gov/hepatitis/statistics/2018surveillance/HepC.htm

4. Alpren C, Dawson EL, John B, et al. Opioid Use Fueling HIV Transmission in an Urban Setting: An Outbreak of HIV Infection Among People Who Inject Drugs—Massachusetts, 2015–2018. *Am J Public Health*. 2019;110(1):37-44. doi:10.2105/AJPH.2019.305366

5. Colwell J. Drug-associated endocarditis on the rise. American College of Physicians. Accessed February 8, 2021. https://acphospitalist.org/archives/2017/10/drug-associated-endocarditis.htm

6. Drug Overdose Mortality by State. Published February 12, 2021. Accessed March 13, 2021. https://www.cdc.gov/nchs/pressroom/sosmap/drug_poisoning_mortality/drug_poisoning.htm

7. Meier A, Moore S, Saunders E, et al. *Hotspot Report: Understanding Opiod Overdoses in New Hampshire*. National Institute on Drug Abuse; 2018. Accessed March 13, 2020. https://umd.app.box.com/v/NDEWS-HotSpot-Report-June-2017

8. Romo E, Wilson D, Stopka TJ, Drew A, Hoskinson, Jr R, de Gijsel D, Marsh BJ, Alston WK, Kelso PT, Nolte K, Friedmann PD. Correlates of HCV infection among people who inject drugs in rural New England: preliminary results from the DISCERNNE study. In: Garets M, Archer S, Kitchens C, Cochran G, Gordon AJ. The 2019 Addiction Health Services Research Conference: Insights, review, and abstracts. Subst Abus. 2019;40(4):469-472 (Supp 203). PMID: 31847783.

9. Hepatitis C Questions and Answers for Health Professionals. Centers for Disease Control and Prevention. Published January 13, 2020. Accessed March 19, 2020. https://www.cdc.gov/hepatitis/hcv/hcvfaq.htm

10. *National Survey of Substance Abuse Treatment Services: State Profiles 2019*. Substance Abuse and Mental Health Services Administration; 2020:324. Accessed February 25, 2021. https://www.samhsa.gov/data/sites/default/files/reports/rpt29397/2019_NSSATS_StPro_combined.pdf

11. *Syringe Services Programs in New Hampshire: State Fiscal Year 2019 Annual Report*. New Hampshire Division of Public Health Services; 2019. Accessed March 14, 2020. https://www.dhhs.nh.gov/dphs/bchs/std/documents/sspreport-2018-2019.pdf

12. Hepatitis C: State of Medicaid Access Report Card. New Hampshire. Hepatitis C: State of Medicaid Access. Published 2017. Accessed August 12, 2020. https://stateofhepc.org/report/#NewHampshire

13. Waters P, Greenwald R, Ninburg M, Simmons A. State Policies Limiting Progress Towards HCV Elimination in the U.S. Presented at the: AASLD The Liver Meeting; November 13, 2020. Accessed February 2, 2021. http://stateofhepc.org/wp-content/uploads/2020/11/AASLD-Poster-NVHR_CHLPI-2020.pdf

14. Blackburn NA, Patel RC, Zibbell JE. Improving Screening Methods for Hepatitis C Among People Who Inject Drugs: Findings from the HepTLC Initiative, 2012-2014. *Public Health Rep Wash DC 1974*. 2016;131 Suppl 2:91-97. doi:10.1177/00333549161310S214

15. Morris MD, Mirzazadeh A, Evans JL, et al. Treatment cascade for hepatitis C virus in young adult people who inject drugs in San Francisco: Low number treated. *Drug Alcohol Depend*. 2019;198:133-135. doi:10.1016/j.drugalcdep.2019.02.008

16. Tsui JI, Miller CM, Scott JD, Corcorran MA, Dombrowski JC, Glick SN. Hepatitis C Continuum of Care and Utilization of Healthcare and Harm Reduction Services among Persons who Inject Drugs in Seattle. *Drug Alcohol Depend*. 2019;195:114-120. doi:10.1016/j.drugalcdep.2018.11.026

17. Chhatwal J, Sussman NL. Universal Screening for Hepatitis C: An Important Step in Virus Elimination. *Clin Gastroenterol Hepatol*. 2019;17(5):835-837. doi:10.1016/j.cgh.2018.12.002

18. Dore GJ, Trooskin S. People with Hepatitis C Who Inject Drugs — Underserved, Not Undeserving. *N Engl J Med*. 2020;383(7):608-611. doi:10.1056/NEJMp2002126

19. Sutherland P. Just Days After Bill's Passage, Needle Exchange Program Up and Running in N.H. New Hampshire Public Radio. Published June 28, 2017. Accessed August 8, 2020. https://www.nhpr.org/post/just-days-after-bills-passage-needle-exchange-program-and-running-nh

20. Fernández-Viña MH, Prood NE, Herpolsheimer A, Waimberg J, Burris S. State Laws Governing Syringe Services Programs and Participant Syringe Possession, 2014-2019. *Public Health Rep*. 2020;135(1_suppl):128S-137S. doi:10.1177/0033354920921817

21. Canary L, Hariri S, Campbell C, et al. Geographic Disparities in Access to Syringe Services Programs Among Young Persons With Hepatitis C Virus Infection in the United States. *Clin Infect Dis*. 2017;65(3):514-517. doi:10.1093/cid/cix333

22. Jones CM, Campopiano M, Baldwin G, McCance-Katz E. National and State Treatment Need and Capacity for Opioid Agonist Medication-Assisted Treatment. *Am J Public Health*. 2015;105(8):e55-e63. doi:10.2105/AJPH.2015.302664

23. *National Survey of Substance Abuse Treatment Services: State Profiles 2013*. Substance Abuse and Mental Health Services Administration; 2013:274. Accessed February 25, 2021. https://www.samhsa.gov/data/sites/default/files/n2013_st_profiles.pdf

24. SAMHSA - Substance Abuse and Mental Health Services Administration. SAMHSA - The Substance Abuse Mental Health Services Administration. Accessed May 8, 2021. https://www.samhsa.gov/node

25. *Reportable Communicable Diseases in New Hampshire, 2014-2019 YTD*. New Hampshire Division of Public Health Services Accessed March 14, 2020. https://www.dhhs.nh.gov/dphs/cdcs/documents/monthly.pdf

26. *Reported Chronic and Acute Hepatitis C Virus (HCV) Infections in New Hampshire, 2017-2019*. New Hampshire Department of Health and Human Services; 2021.

27. Stopka TJ, Jacque E, Kelso P, et al. The opioid epidemic in rural northern New England: An approach to epidemiologic, policy, and legal surveillance. *Prev Med*. 2019;128:105740. doi:10.1016/j.ypmed.2019.05.028

28. *New Hampshire Drug Monitoring Initiative*. New Hampshire Department of Health and Human Services; 2019. Accessed September 7, 2020. https://www.dhhs.nh.gov/dcbcs/bdas/data.htm

29. Ward Z, Platt L, Sweeney S, et al. Impact of current and scaled-up levels of hepatitis C prevention and treatment interventions for people who inject drugs in three UK settings—what is required to achieve the WHO's HCV elimination targets? *Addict Abingdon Engl*. 2018;113(9):1727-1738. doi:10.1111/add.14217

© 2021 Blake A et al. *JAMA Network Open.*

30. Heffernan A, Cooke GS, Nayagam S, Thursz M, Hallett TB. Scaling up prevention and treatment towards the elimination of hepatitis C: a global mathematical model. *Lancet Lond Engl*. 2019;393(10178):1319-1329. doi:10.1016/S0140-6736(18)32277-3

31. Scott N, Ólafsson S, Gottfreðsson M, et al. Modelling the elimination of hepatitis C as a public health threat in Iceland: A goal attainable by 2020. *J Hepatol*. 2018;68(5):932-939. doi:10.1016/j.jhep.2017.12.013

32. Mathers BM, Degenhardt L, Bucello C, Lemon J, Wiessing L, Hickman M. Mortality among people who inject drugs: a systematic review and meta-analysis. *Bull World Health Organ*. 2013;91(2):102-123. doi:10.2471/BLT.12.108282

33. Arias E, Xu J, Kochanek K. United States Life Tables, 2016. 2019;68(4):66.

34. Durham DP, Skrip LA, Bruce RD, et al. The Impact of Enhanced Screening and Treatment on Hepatitis C in the United States. *Clin Infect Dis Off Publ Infect Dis Soc Am*. 2016;62(3):298-304. doi:10.1093/cid/civ894

35. Nosyk B, Li L, Evans E, et al. Characterizing longitudinal health state transitions among heroin, cocaine, and methamphetamine users. *Drug Alcohol Depend*. 2014;140:69-77. doi:10.1016/j.drugalcdep.2014.03.029

36. *Key Substance Use and Mental Health Indicators in the United States: Results from the 2018 National Survey on Drug Use and Health*. Substance Abuse and Mental Health Services Administration Accessed July 24, 2020. https://www.samhsa.gov/data/sites/default/files/cbhsq-reports/NSDUHNationalFindingsReport2018/NSDUHNationalFindingsReport2018.pdf

37. Fraser H, Zibbell J, Hoerger T, et al. Scaling-up HCV prevention and treatment interventions in rural United States-model projections for tackling an increasing epidemic. *Addict Abingdon Engl*. 2018;113(1):173-182. doi:10.1111/add.13948

38. Roy E, Boudreau J-F, Boivin J-F. Hepatitis C virus incidence among young street-involved IDUs in relation to injection experience. *Drug Alcohol Depend*. 2009;102(1-3):158-161. doi:10.1016/j.drugalcdep.2009.01.006

39. Platt L, Minozzi S, Reed J, et al. Needle syringe programmes and opioid substitution therapy for preventing hepatitis C transmission in people who inject drugs. *Cochrane Database Syst Rev*. 2017;9:CD012021. doi:10.1002/14651858.CD012021.pub2

40. Kabiri M, Jazwinski AB, Roberts MS, Schaefer AJ, Chhatwal J. The changing burden of hepatitis C virus infection in the United States: model-based predictions. *Ann Intern Med*. 2014;161(3):170-180. doi:10.7326/M14-0095

41. *Treatment Episode Data Set: Admissions (TEDS-A)*. Substance Abuse and Mental Health Services Administration; 2018. Accessed March 13, 2020. https://www.datafiles.samhsa.gov/study-series/treatment-episode-data-set-admissions-teds-nid13518

42. Jones CM. Patterns and Characteristics of Methamphetamine Use Among Adults — United States, 2015–2018. *MMWR Morb Mortal Wkly Rep*. 2020;69. doi:10.15585/mmwr.mm6912a1

43. Novak SP, Kral AH. Comparing Injection and Non-Injection Routes of Administration for Heroin, Methamphetamine, and Cocaine Users in the United States. *J Addict Dis*. 2011;30(3):248-257. doi:10.1080/10550887.2011.581989

44. Han B, Volkow ND, Compton WM, McCance-Katz EF. Reported Heroin Use, Use Disorder, and Injection Among Adults in the United States, 2002-2018. *JAMA*. 2020;323(6):568-571. doi:10.1001/jama.2019.20844

© 2021 Blake A et al. *JAMA Network Open.*

45. Vital Statistics Rapid Release - Provisional Drug Overdose Data. Centers for Disease Control and Prevention. Published April 7, 2021. Accessed May 11, 2021. https://www.cdc.gov/nchs/nvss/vsrr/drug-overdose-data.htm

46. Rosenberg ES, Rosenthal EM, Hall EW, et al. Prevalence of Hepatitis C Virus Infection in US States and the District of Columbia, 2013 to 2016. *JAMA Netw Open*. 2018;1(8):e186371. doi:10.1001/jamanetworkopen.2018.6371

47. MappingHepC. MappingHepC. Published July 2020. Accessed March 22, 2021. https://mappinghepc.com/maps

48. Chirikov VV, Marx SE, Manthena SR, Strezewski JP, Saab S. Development of a Comprehensive Dataset of Hepatitis C Patients and Examination of Disease Epidemiology in the United States, 2013-2016. Adv Ther 2018;35:1087–1102.

49. Klevens RM, Liu S, Roberts H, Jiles RB, Holmberg SD. Estimating acute viral hepatitis infections from nationally reported cases. *Am J Public Health*. 2014;104(3):482-487. doi:10.2105/AJPH.2013.301601

eFigure 1. Drug Overdose Rates

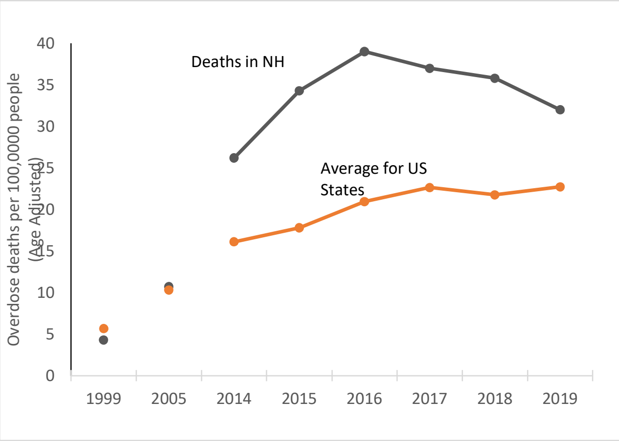

a) New Hampshire and US average (Data from the CDC.[6])

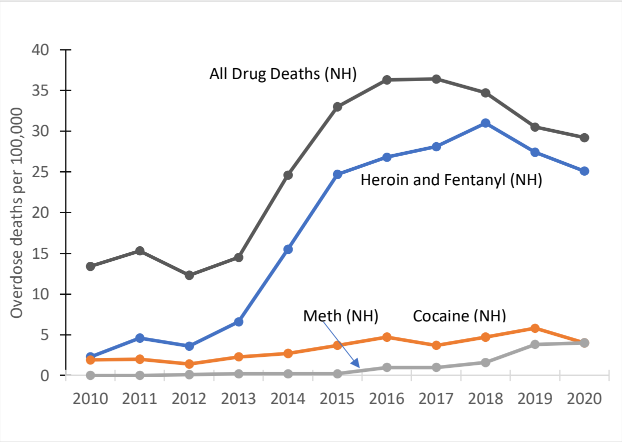

b) Overdose deaths by drug in New Hampshire (Data from NH DMI[2])

© 2021 Blake A et al. *JAMA Network Open.*

eFigure 2. Injector Duration Submodel Structure

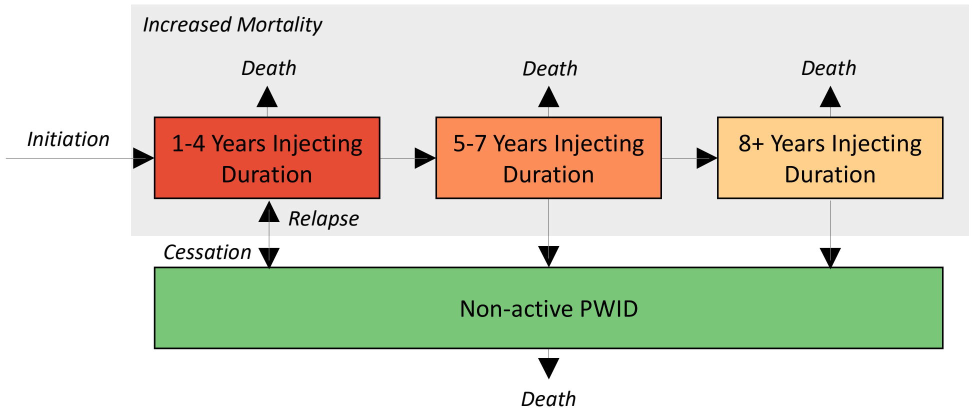

© 2021 Blake A et al. *JAMA Network Open.*

eFigure 3. MAT/SSP Enrollment Submodel Structure

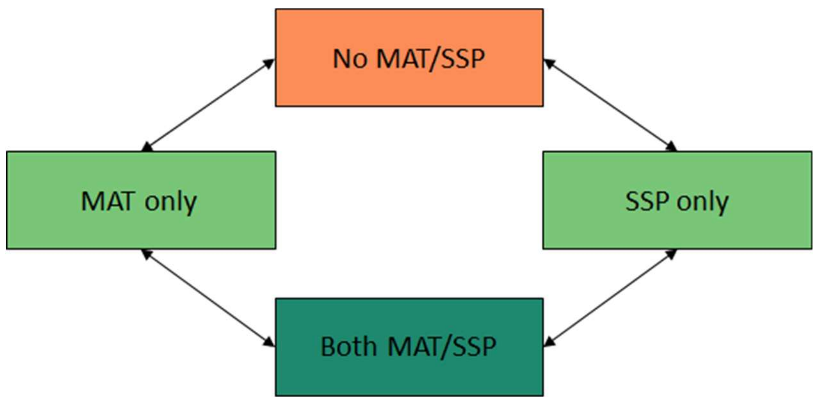

eFigure 4. Model Forecasts for Scenario 1, Base Case

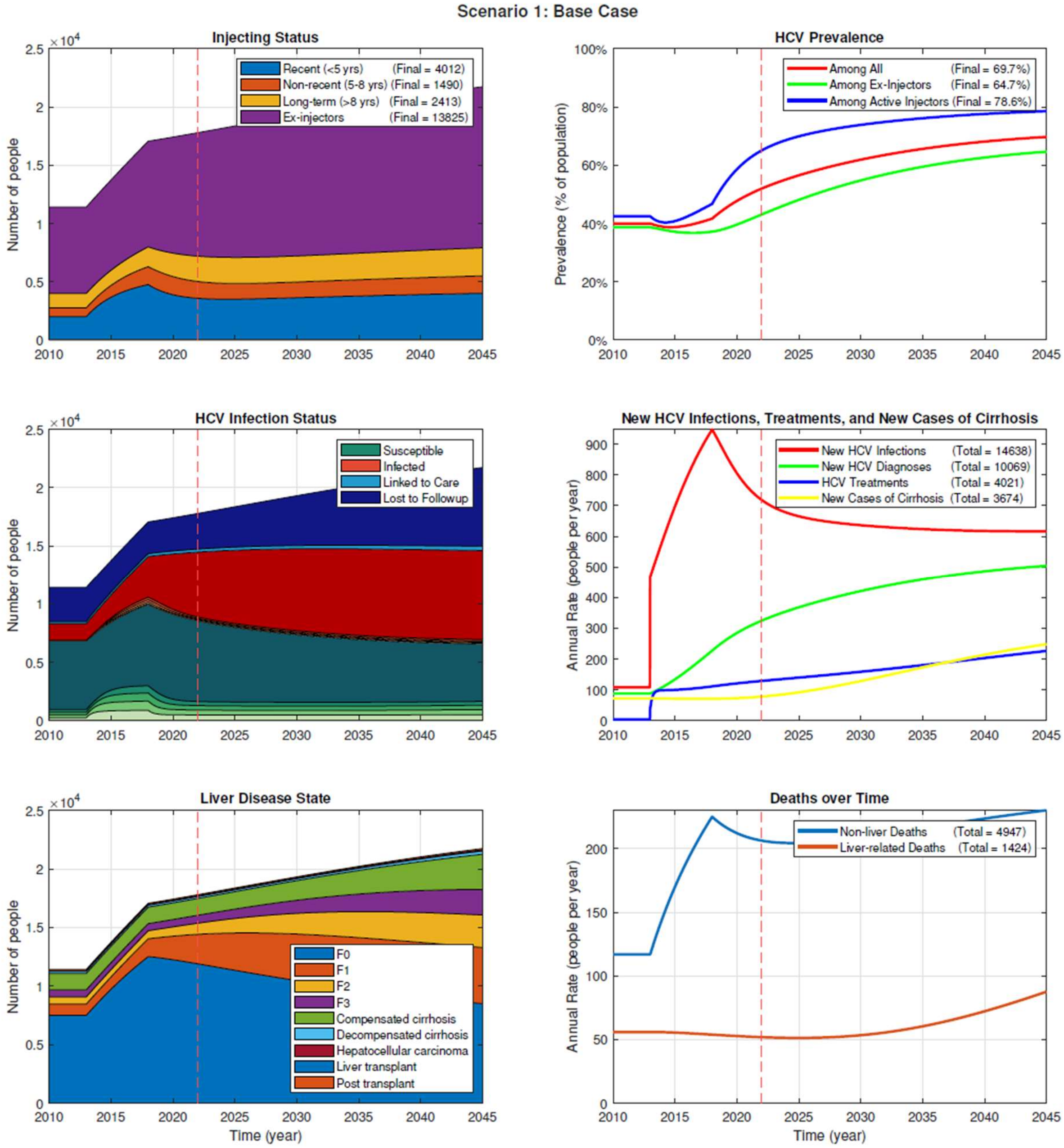

© 2021 Blake A et al. *JAMA Network Open.*

eFigure 5. Model Forecasts for Scenario 2, Improved Harm Reduction

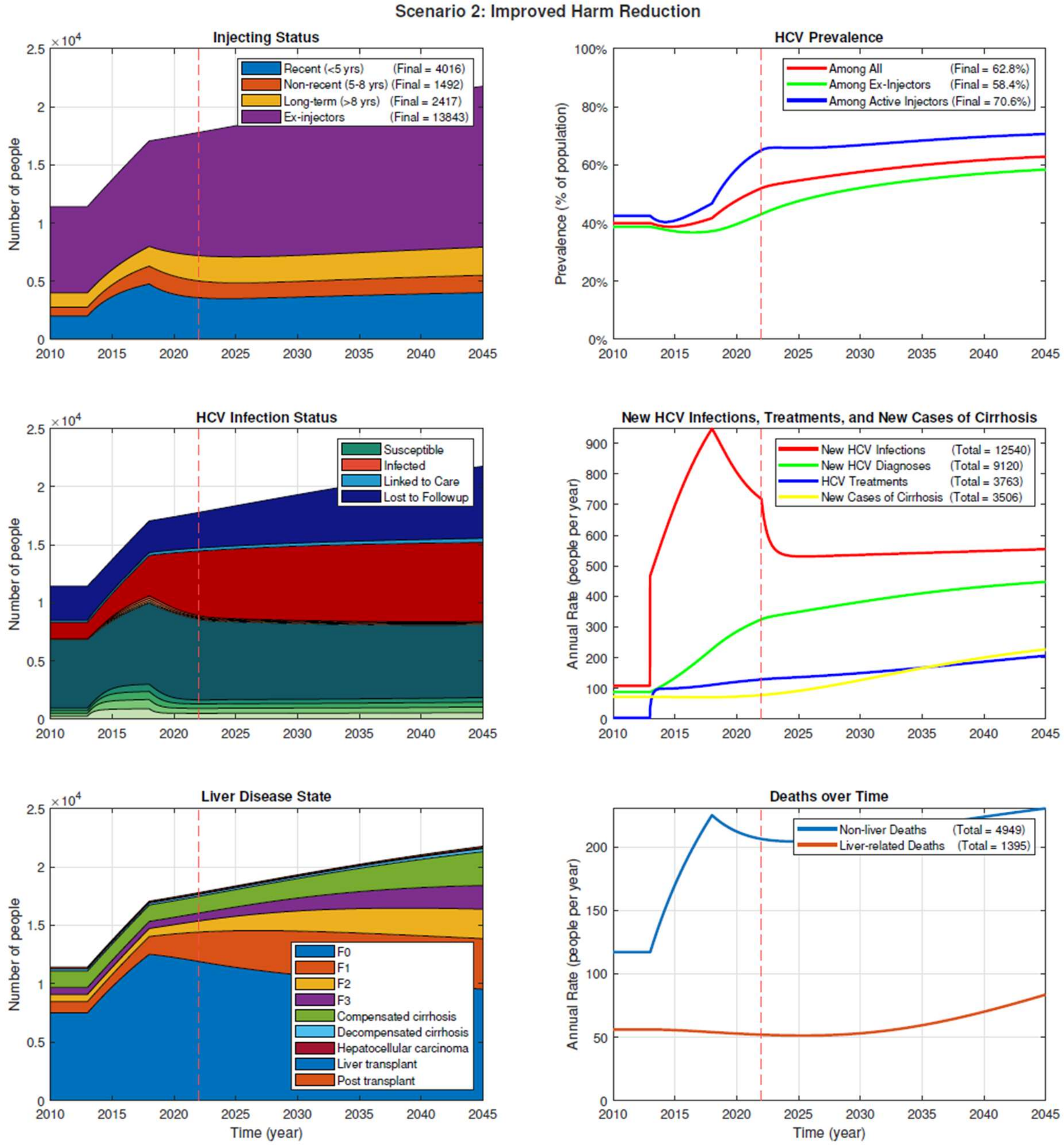

© 2021 Blake A et al. *JAMA Network Open.*

eFigure 6. Model Forecasts for Scenario 3, Improved Testing

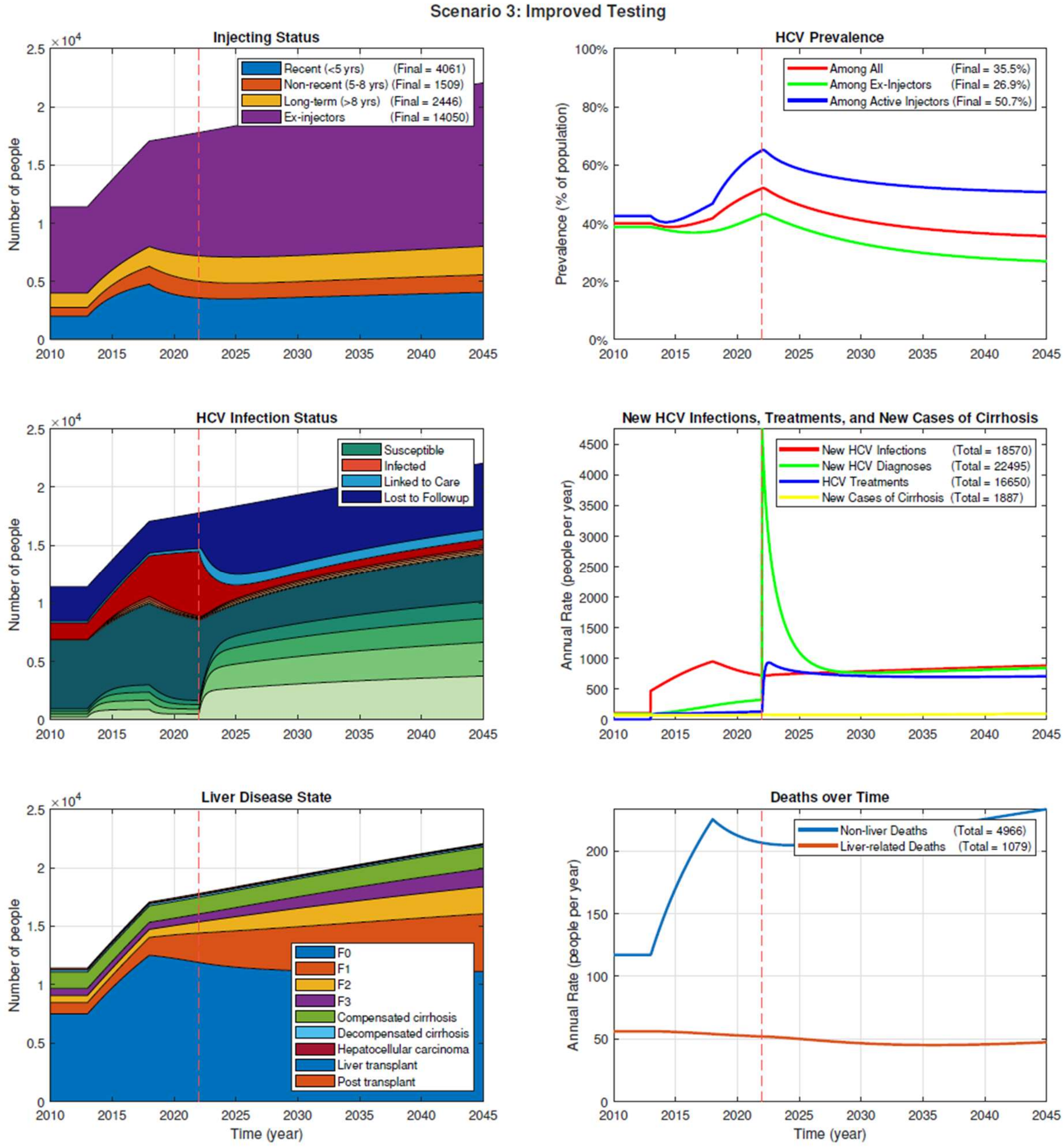

© 2021 Blake A et al. *JAMA Network Open.*

eFigure 7. Model Forecasts for Scenario 4, Improved Treatment

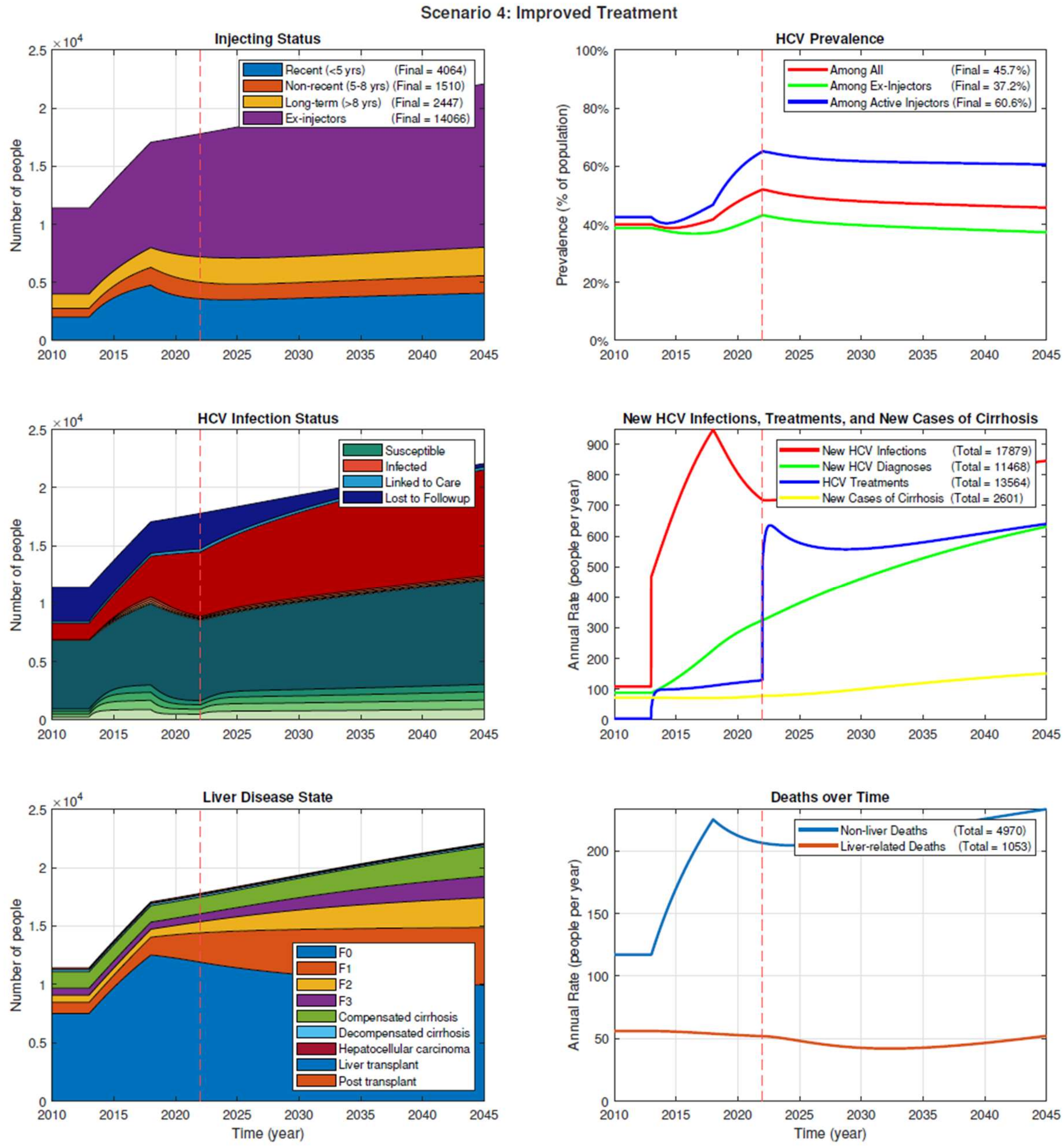

© 2021 Blake A et al. *JAMA Network Open.*

eFigure 8. Model Forecasts for Scenario 5, Improved Testing and Treatment

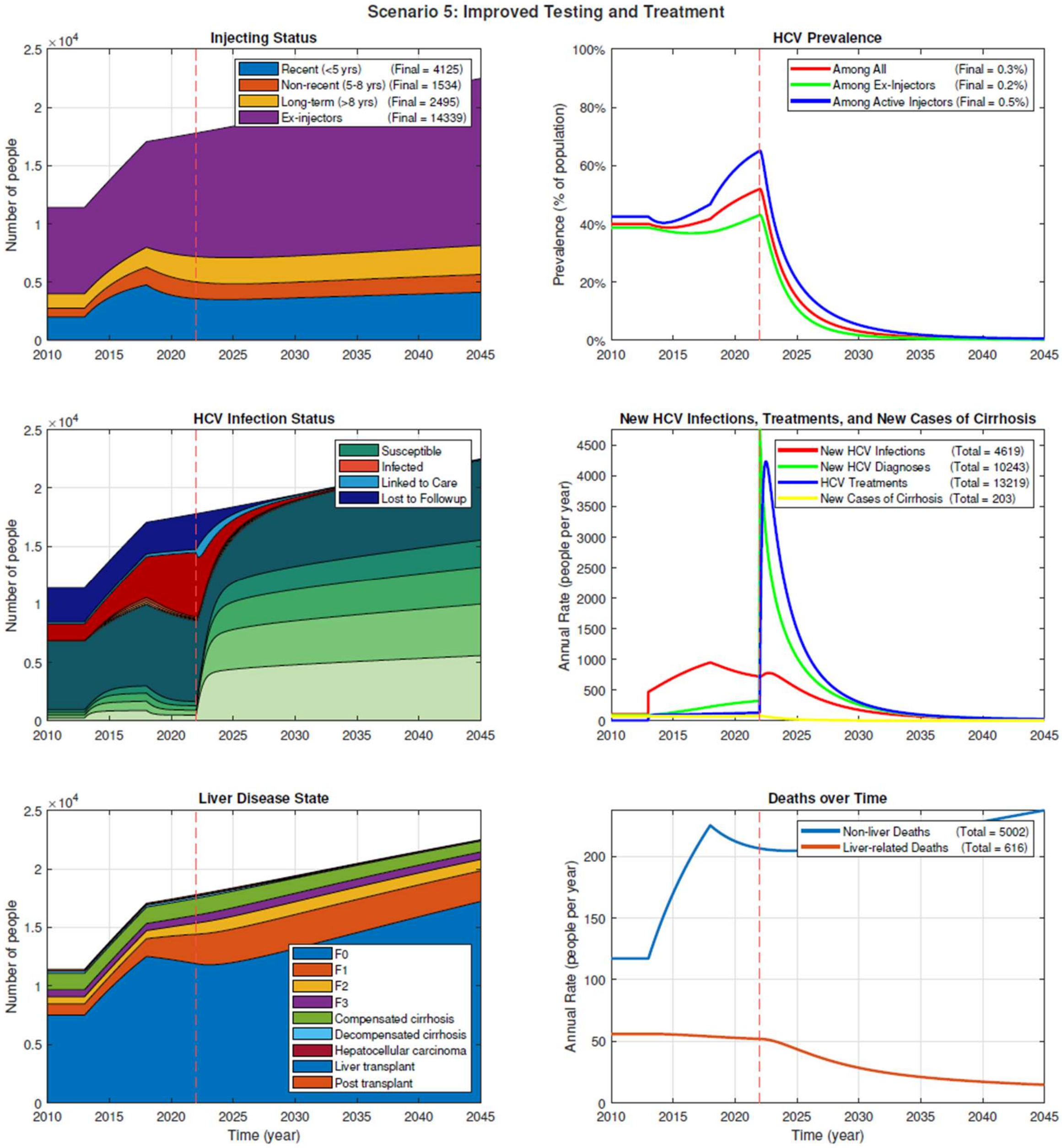

© 2021 Blake A et al. *JAMA Network Open.*

eFigure 9. Model Forecasts for Scenario 6, Improved Testing, Treatment and Harm Reduction

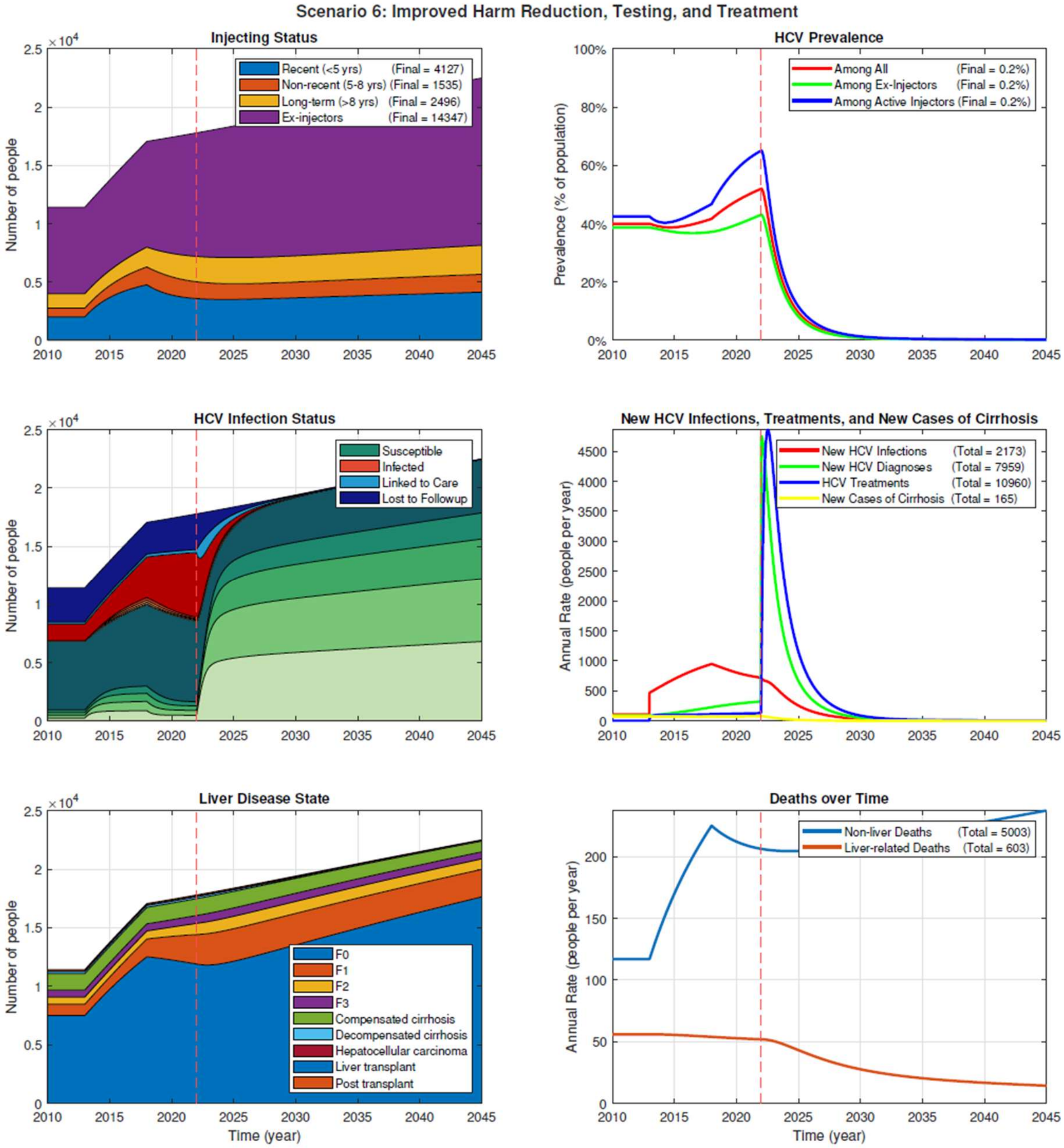

© 2021 Blake A et al. *JAMA Network Open.*

eFigure 10. Policy Intervention Sensitivity Analysis

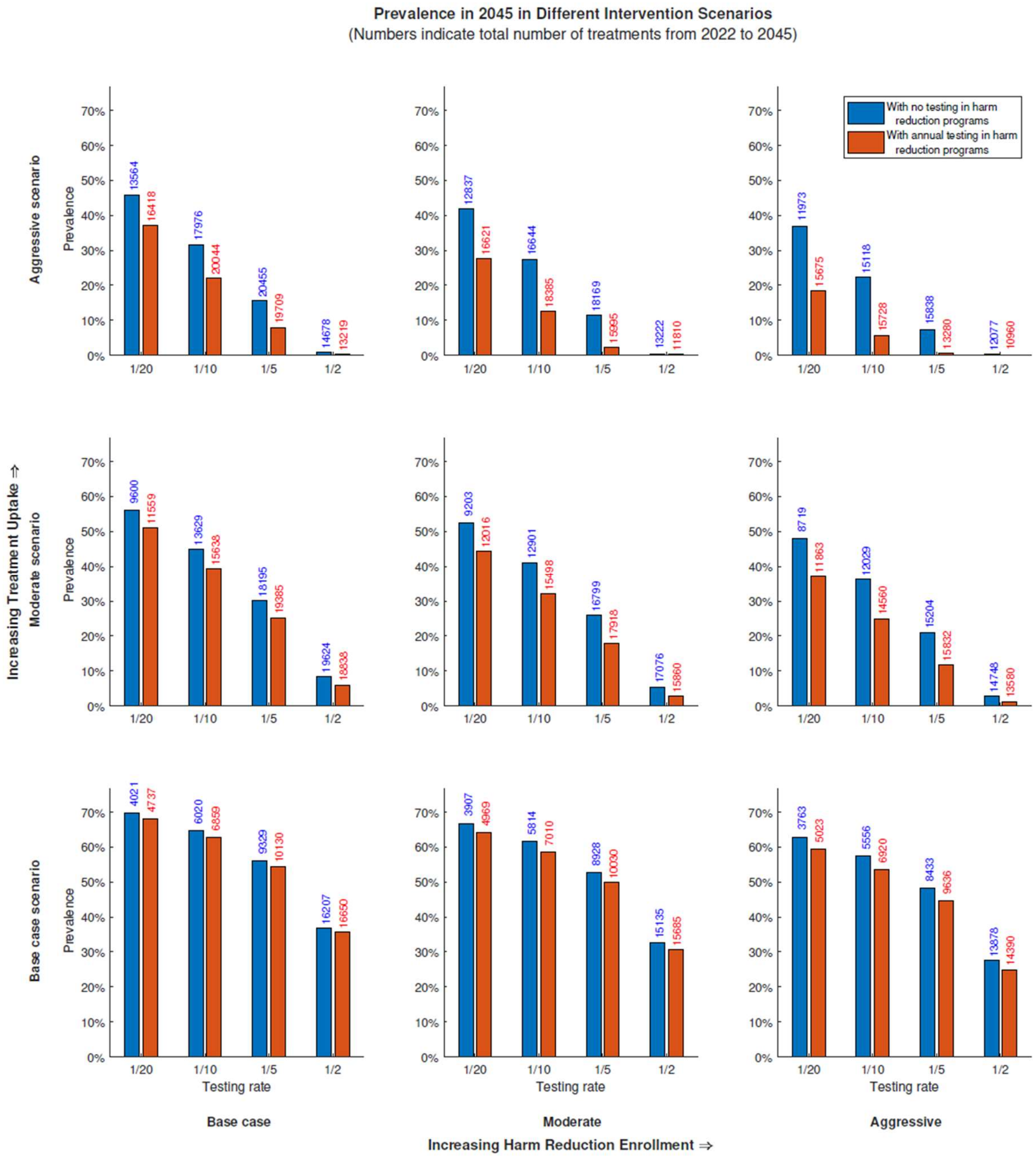

© 2021 Blake A et al. *JAMA Network Open.*

eFigure 11. Simulation Results for Scenario 1, Base Case

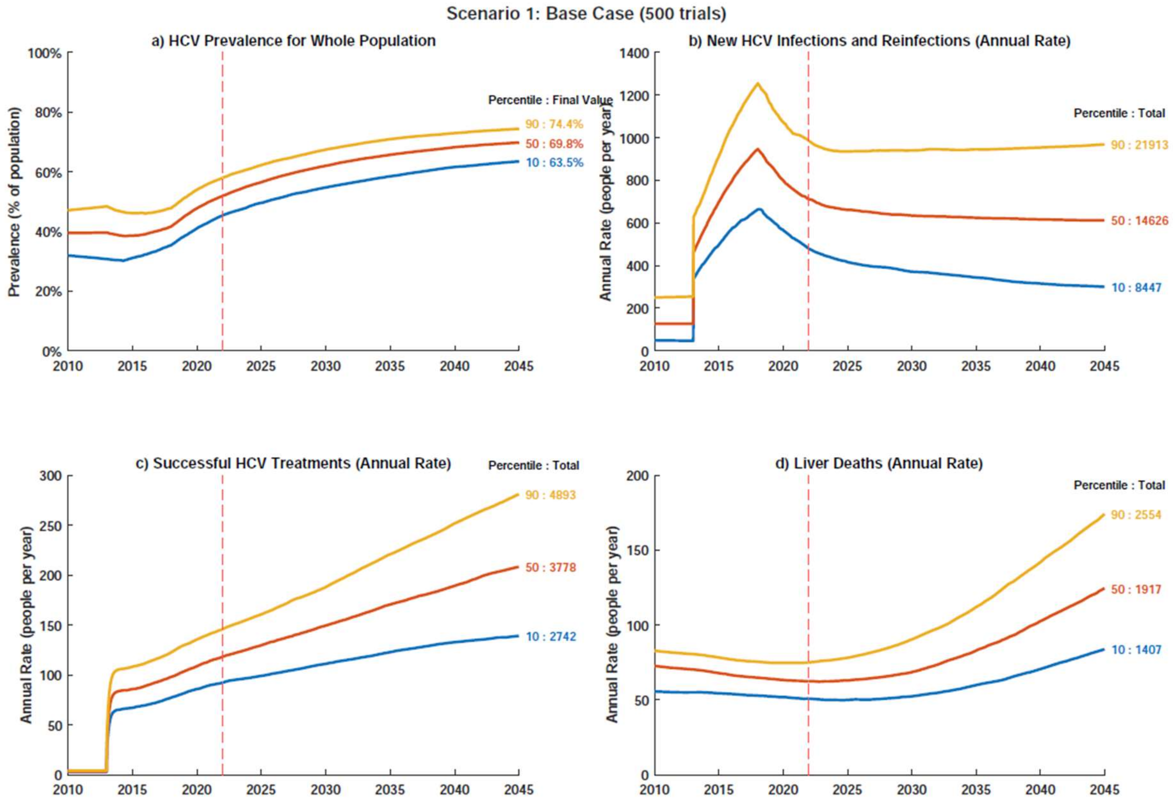

© 2021 Blake A et al. *JAMA Network Open.*

eFigure 12. Simulation Results for Scenario 2, Increased Harm Reduction

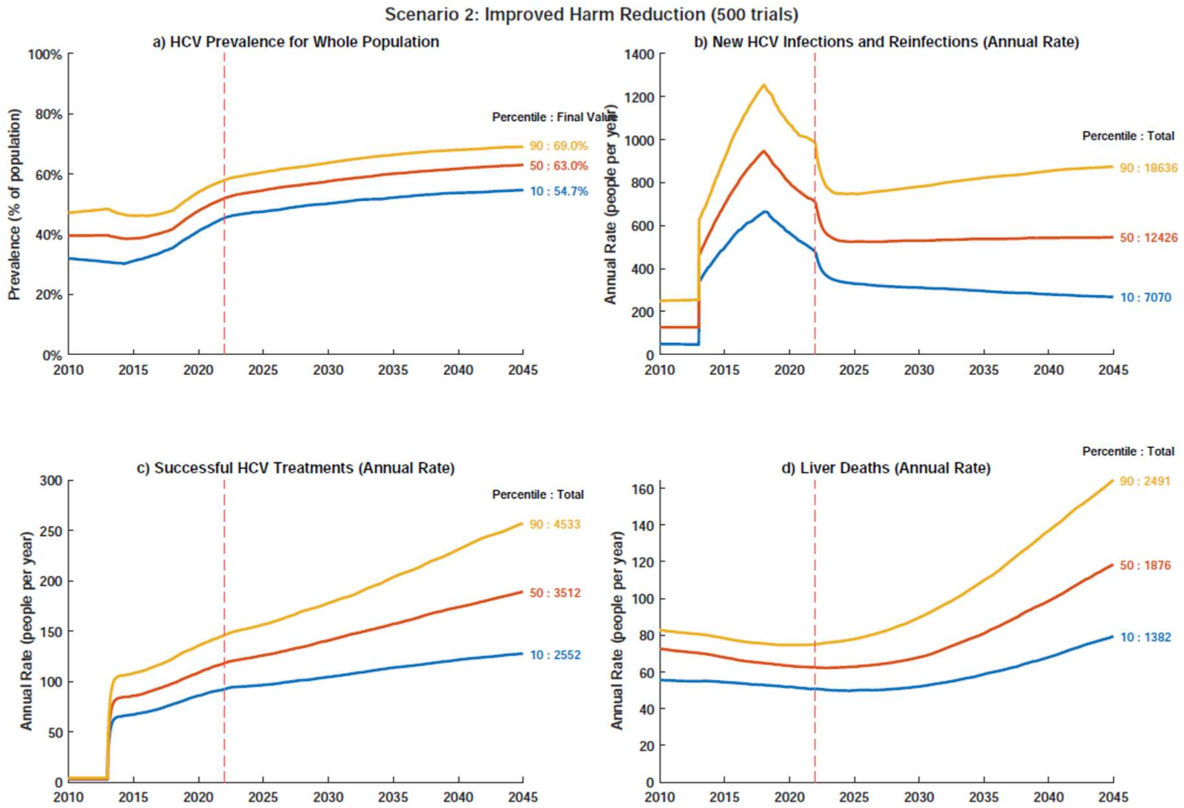

© 2021 Blake A et al. *JAMA Network Open.*

eFigure 13. Simulation Results for Scenario 3, Improved Testing

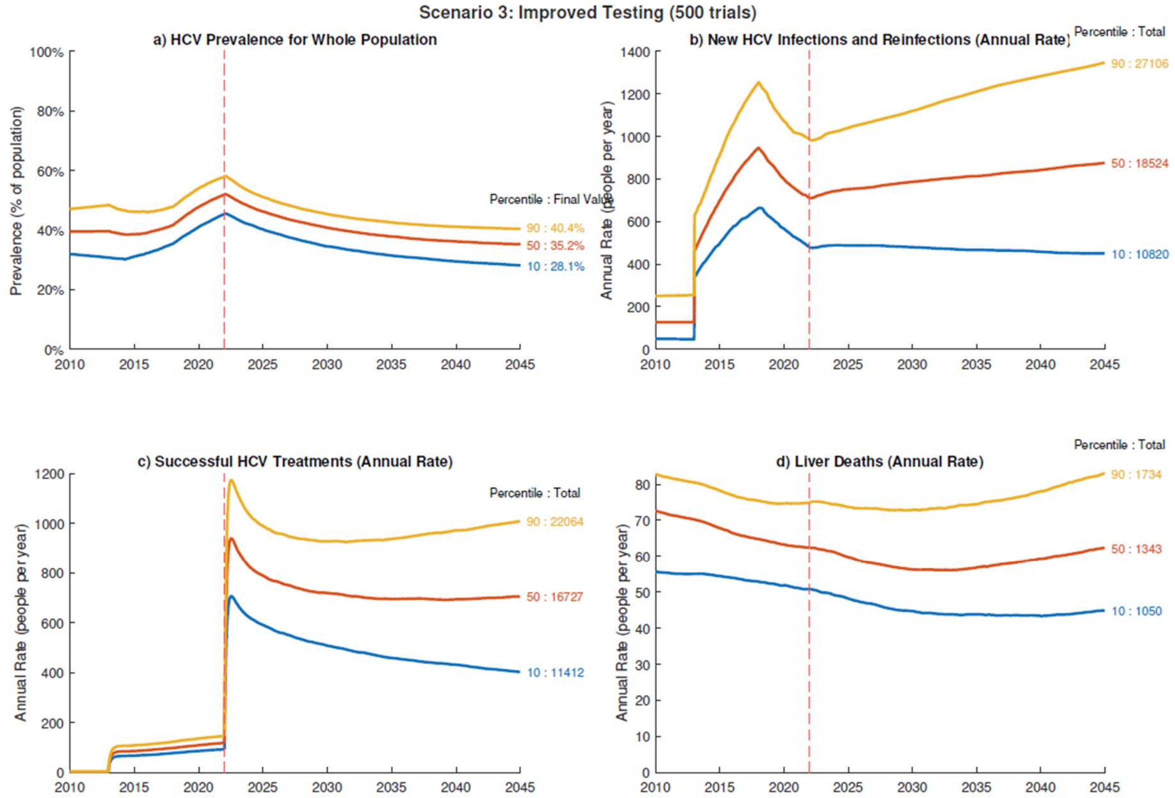

© 2021 Blake A et al. *JAMA Network Open.*

eFigure 14. Simulation Results for Scenario 4, Improved Treatment

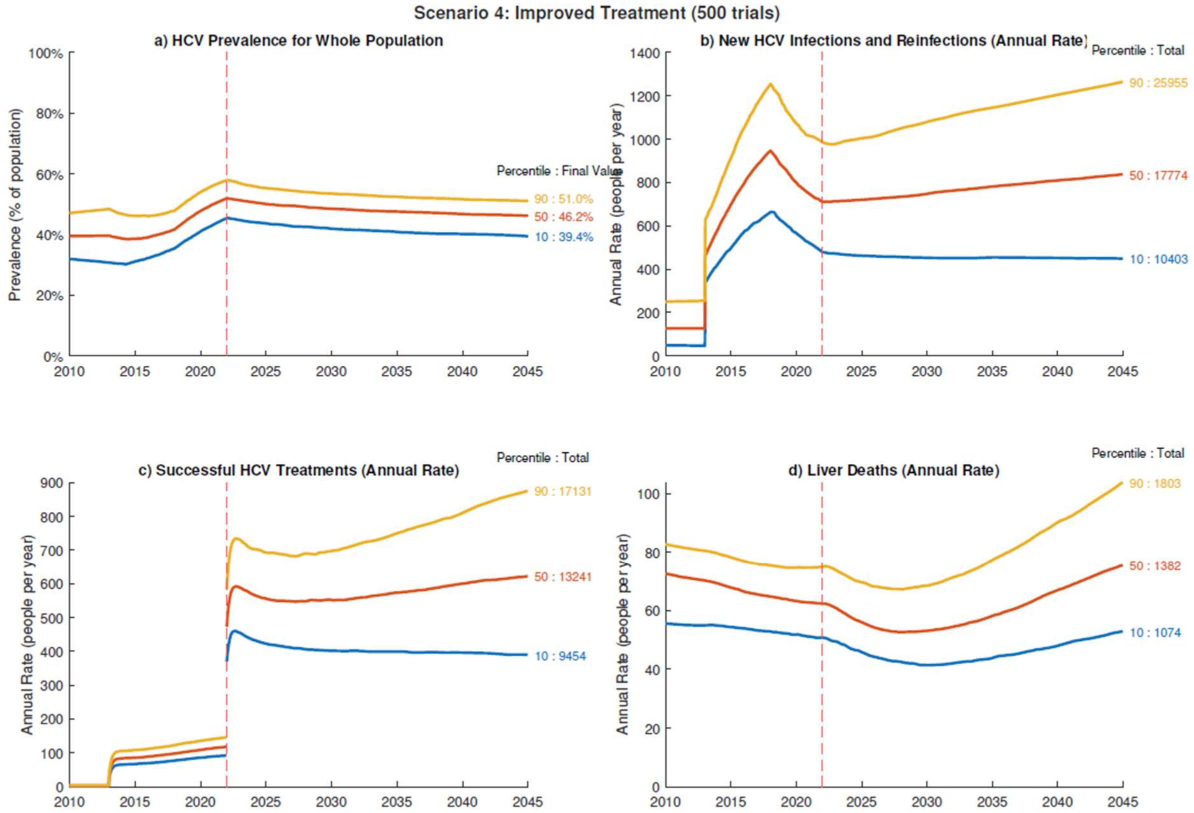

© 2021 Blake A et al. *JAMA Network Open.*

eFigure 15. Simulation Results for Scenario 5, Improved Testing and Treatment

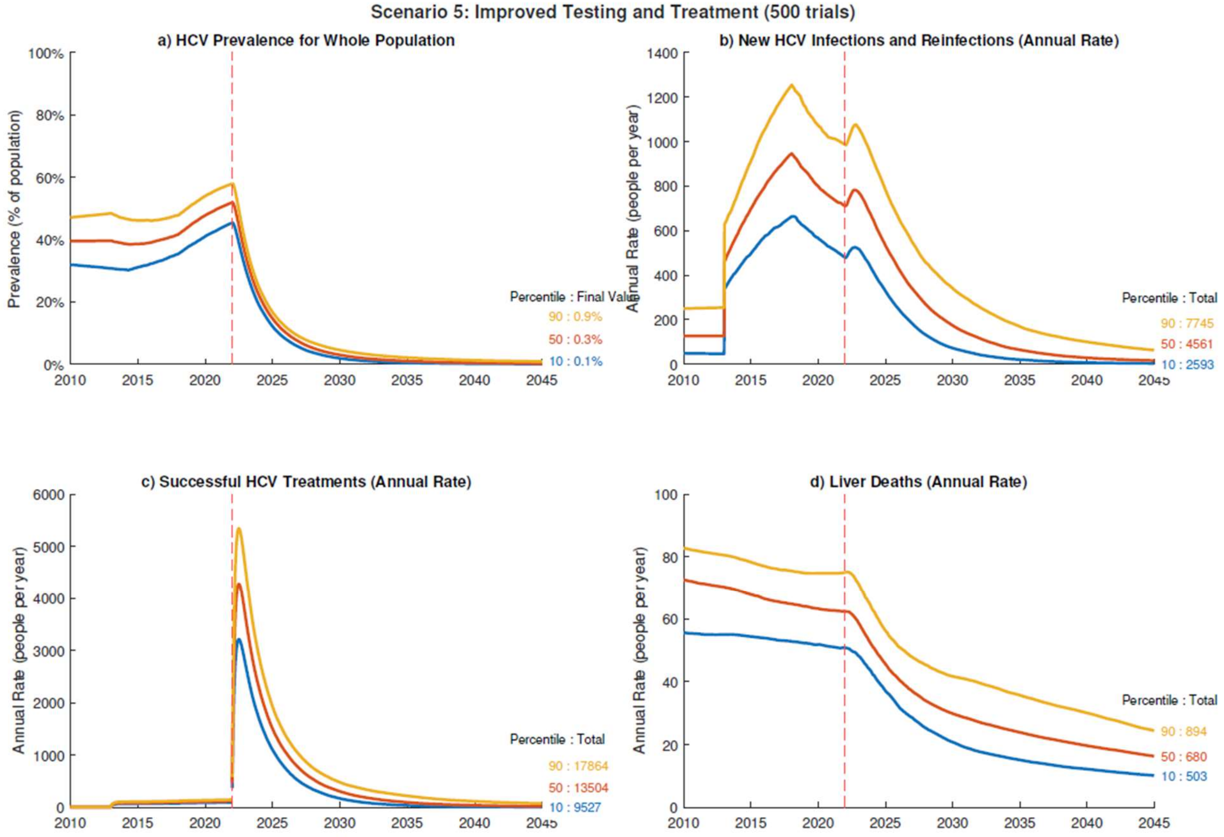

© 2021 Blake A et al. *JAMA Network Open.*

eFigure 16. Simulation Results for Scenario 6, Improved Testing, Treatment, and Harm Reduction

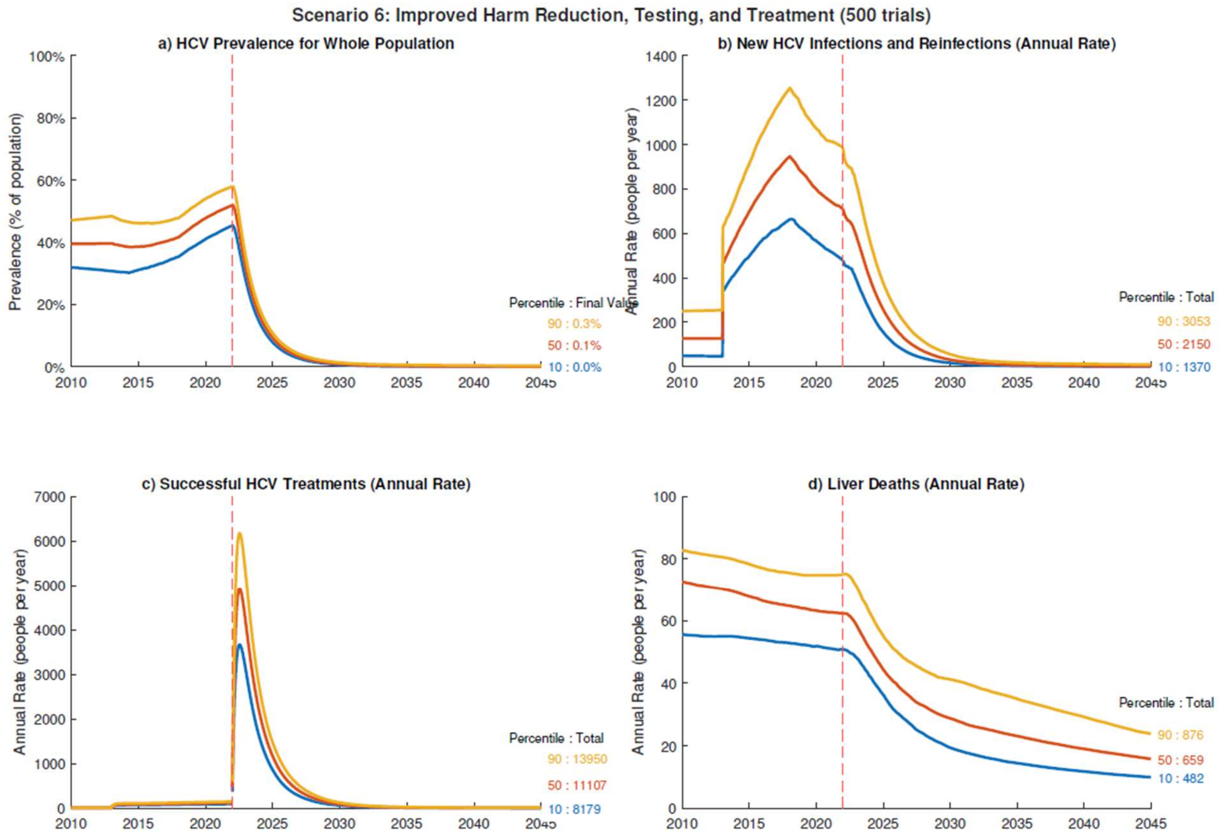

eFigure 17. Sensitivity Analysis to Limit on Treatment Rate in Scenario 6

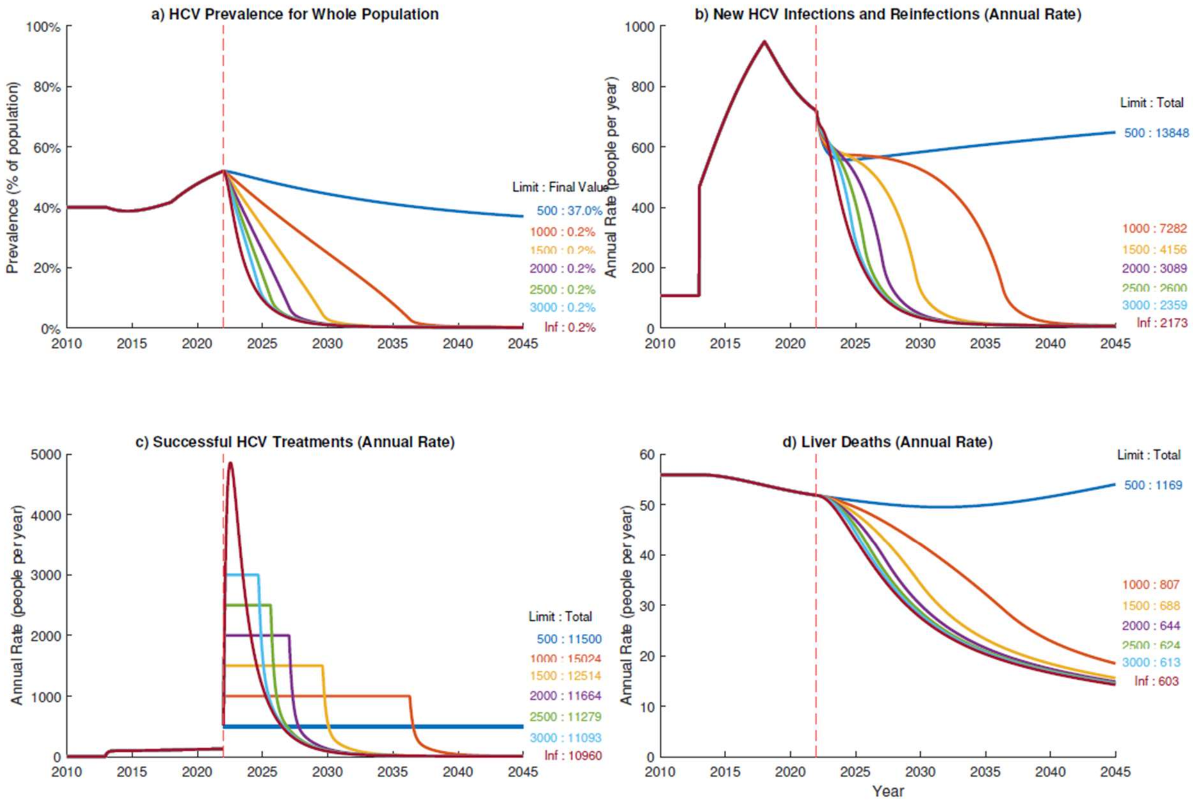

© 2021 Blake A et al. *JAMA Network Open.*
